# Diastereoselective synthesis of novel (*S*)-lactic pyrazoline derivatives and investigation of antibacterial capabilities

**DOI:** 10.1038/s41598-025-32881-3

**Published:** 2025-12-21

**Authors:** Maryam Gholami, Gholamhassan Imanzadeh, Farhad Kabiri Esfahani, Morteza Karami-Zarandi, Amir Nasser Shamkhali

**Affiliations:** 1 Department of Chemistry, Faculty of Sciences, University of Mohaghegh Ardabili, Ardabil, 56199-11367 Iran; 2 Department of Chemistry, Faculty of Sciences, University of Zanjan, Zanjan, 45371-38791 Iran; 3 Department of Microbiology, School of Medicine, Zanjan University of Medical Sciences, Zanjan, 45139-56111 Iran

**Keywords:** Diastereoselective synthesis, (*S*)-lactic pyrazoline, (*S*)-lactic hydrazide, Chirality induction, Antibacterial properties, Chemistry, Drug discovery

## Abstract

In this study, a novel series of diastereomerically enriched lactic-derived pyrazoline compounds **7(a–l)** were synthesized via cyclocondensation of (*S*)-lactic Hydrazide with various substituted Chalcones under basic conditions. The methodology benefits from the inherent chirality of (*S*)-ethyl lactate, serving as a chiral precursor, allowing diastereoselectivity in the formation of (*S*,*R*)-pyrazoline derivatives. Structural confirmation was achieved using IR, ^1^H NMR, ^13^C NMR, mass spectroscopic data, and elemental analysis. The proposed stereochemistry of compound **7b** has been further corroborated by X-ray diffraction study. This selectivity can be related to the stability of (*S*,*R*)-pyrazolines versus (*S*,*S*)-pyrazoline derivatives, that our DFT calculation method confirmed this idea. The yields of the target compounds ranged from 24 to 63%, with approximately 100% diastereoselectivity giving exclusively the corresponding diastereoisomers that have (*R*)-configuration of the chiral carbon in their pyrazoline ring moiety. Evaluation of the antibacterial activity revealed that compounds **7g** (IZD: 12 mm, MIC: 264 µg/ml) and **7j** (IZD: 14 mm, MIC: 264 µg/ ml) exhibited considerable effect against Gram-positive *S. aureus*. Additionally, compound **7h** (IZD: 10 mm, MIC: 512 µg/ml) demonstrated activity against *P. aeruginosa* and *A. baumannii*.

**Supplementary Information:**

The online version contains supplementary material available at 10.1038/s41598-025-32881-3.

## Introduction

Pyrazolines are versatile compounds in various applications such as fluorescent probes in some elaborate chemosensors, hole-transport material, brightening agents in synthetic fibers, papers, and textiles, recognition of transition metal ions, electroluminescence, electrophotography, and organic electronics^1–11^. Furthermore, the Pyrazoline scaffold is present in the structure of various marketed drug agents of different categories^12^. These compounds have various and impressive biological activities, such as anti-cancer^13^, anti-bacterial^14^, anti-fungal^15^, anti-HIV^16^, anti-tubercular^17^, anti-oxidant^18^, anti-malaria^19^, anti-viral^20^, and many others^21^.

There are indeed several diverse synthetic protocols for the synthesis of pyrazolines and their derivatives. The most common and important synthetic approaches include cyclization of chalcones with hydrazine hydrate and derivatives^22,23^. Cyclization of hydrazones^24^, utilizing hydrazines^25^, Huisgen zwitterion^26^, diazo compounds^27^, nitrile imine^28^, and miscellaneous^29^.

Chiral compounds containing multiple stereogenic centers are commonly found in natural products and pharmaceutical agents^30^. In this regard, numerous diastereoselective approaches have been reported for the asymmetric synthesis of pyrazoline derivatives. These include a green, solvent-free three-component reaction catalyzed by a recyclable Brønsted acidic ionic liquid between aldehydes, hydrazines, and dimethyl acetylenedicarboxylate (DMAD)^31^, [3 + 2] cycloadditions utilizing Baylis–Hillman adducts and nitrilimines^32^, 1,3-dipolar cycloadditions involving diazoalkanes and electron-deficient alkenes^33^, substrate-controlled cycloadditions mediated by tris(dimethylamino)phosphine between azoalkenes and α-dicarbonyl compounds^34^, and high-yielding [3 + 2] cycloadditions between N-vinyl-chalcone-derived nitrones and hydrazonoyl chlorides^35^. Most reported methods for synthesizing optically active pyrazolines, despite their potential utility, are often limited by several drawbacks. They often need costly chiral catalysts or materials, take a long time to react, use harmful organic solvents, and require a lot of catalysts to get the right stereoselectivity. This leads to serious economic and safety issues. Therefore, the use of a simple, efficient, cost-effective, and green method for the diastereoselective synthesis of pyrazolines is highly desirable. The importance and widespread application of the pyrazoline scaffold and its derivatives, particularly in the pharmaceutical field, interested us in joining these research lines aimed at asymmetric synthesis of a new series of its diastereomeric derivatives. In doing so, we employed the inexpensive (*S*)-lactic hydrazide as the chiral building block. This strategy takes advantage of the inherent chirality of a natural, bio-based precursor to induce chirality, enabling the asymmetric synthesis of diastereomerically enriched pyrazoline derivatives through a simpler, greener, and more cost-effective alternative to previously reported.

## Results and discussion

**Scheme 1:**
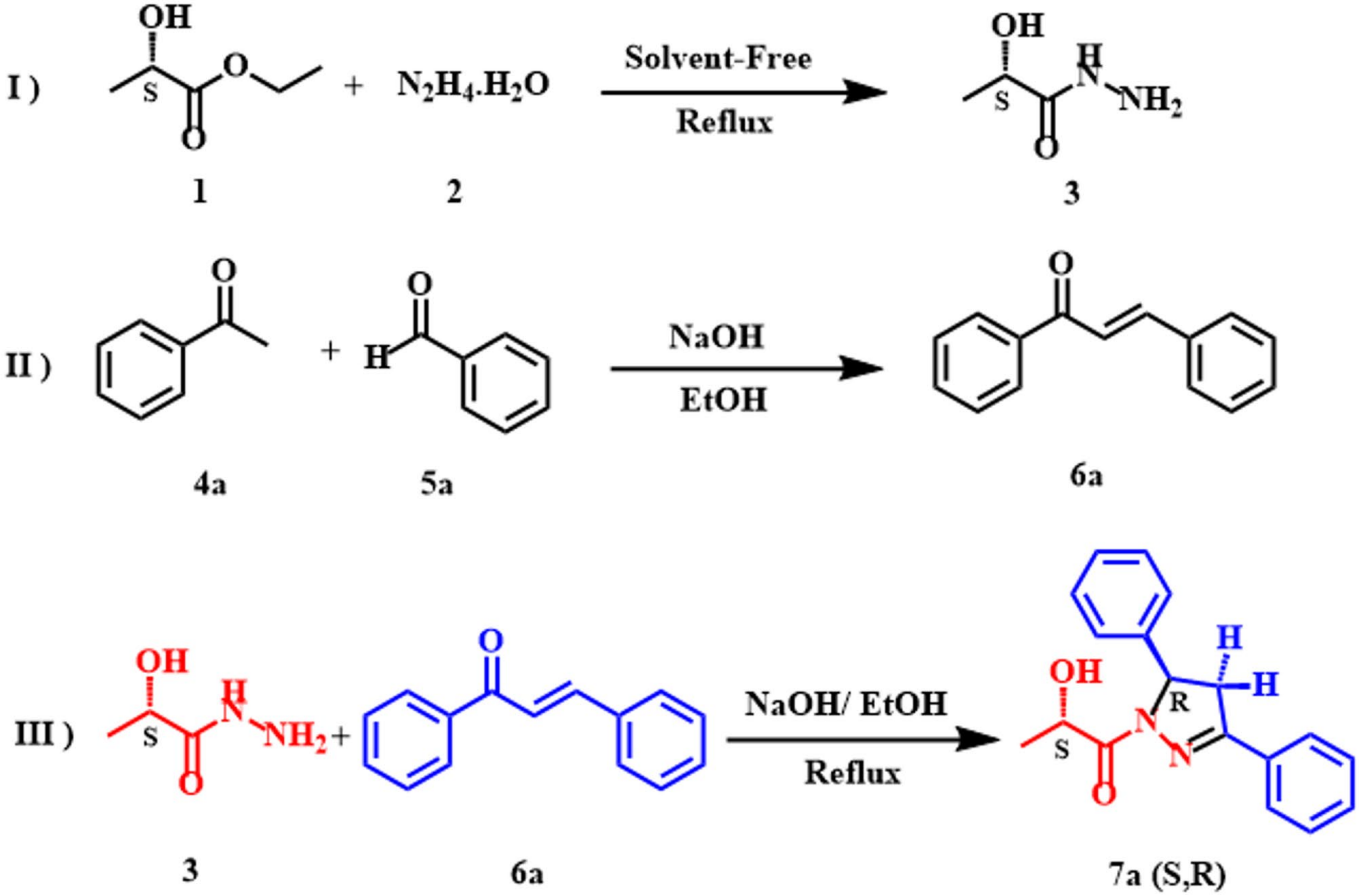
Protocol for the synthesis of lactic-pyrazoline derivatives. Reaction conditions: Ⅰ) **1** (10 mmol), **2** (10 mmol), Reflux, 3 h. Ⅱ) **4a** (6 mmol), **5a** (6 mmol), EtOH (20 ml), NaOH(aq 40%) dropwise in 10 min and stirrer at 0–10 °C 1 h, stirrer at r.t 4 h. Ⅲ) **3** (1 mmol), (1 mmol), **6a** (1 mmol), EtOH (10 ml), NaOH (0.5 mmol), Reflux, 24 h.

The reaction between **3** and chalcone **6a** performed as a model reaction (Scheme 1). Initially, (*S*)-ethyl lactate **1** undergoes a solvent-free reaction with hydrazine hydrate **2** to produce lactic hydrazide **3** (Scheme 1. Reaction Ⅰ). The aldehyde **5a** was reacted with acetophenone **4a** by Claisen-Schmidt reaction in the presence of sodium hydroxide in ethanol to obtain chalcone **6a** with high yield (Scheme 1. Reaction Ⅱ). The reaction of hyrazide **3** with **6a** compound led to pyrazoline **7a** as a single product (Scheme 1. Reaction Ⅲ). Optimization of this reaction (c) conditions was performed by varying the amount of sodium hydroxide, solvent, and reaction time. The best conditions were obtained when 50 mol% sodium hydroxide was used in ethanol with 24 h reaction time. Even with increasing the amount of sodium hydroxide as well as the reaction time, there was no observed improvement in the reaction performance. The structure of this adduct was elucidated using ^1^H, ^13^C NMR, IR, and Mass spectroscopic data.

**Fig. 1:**
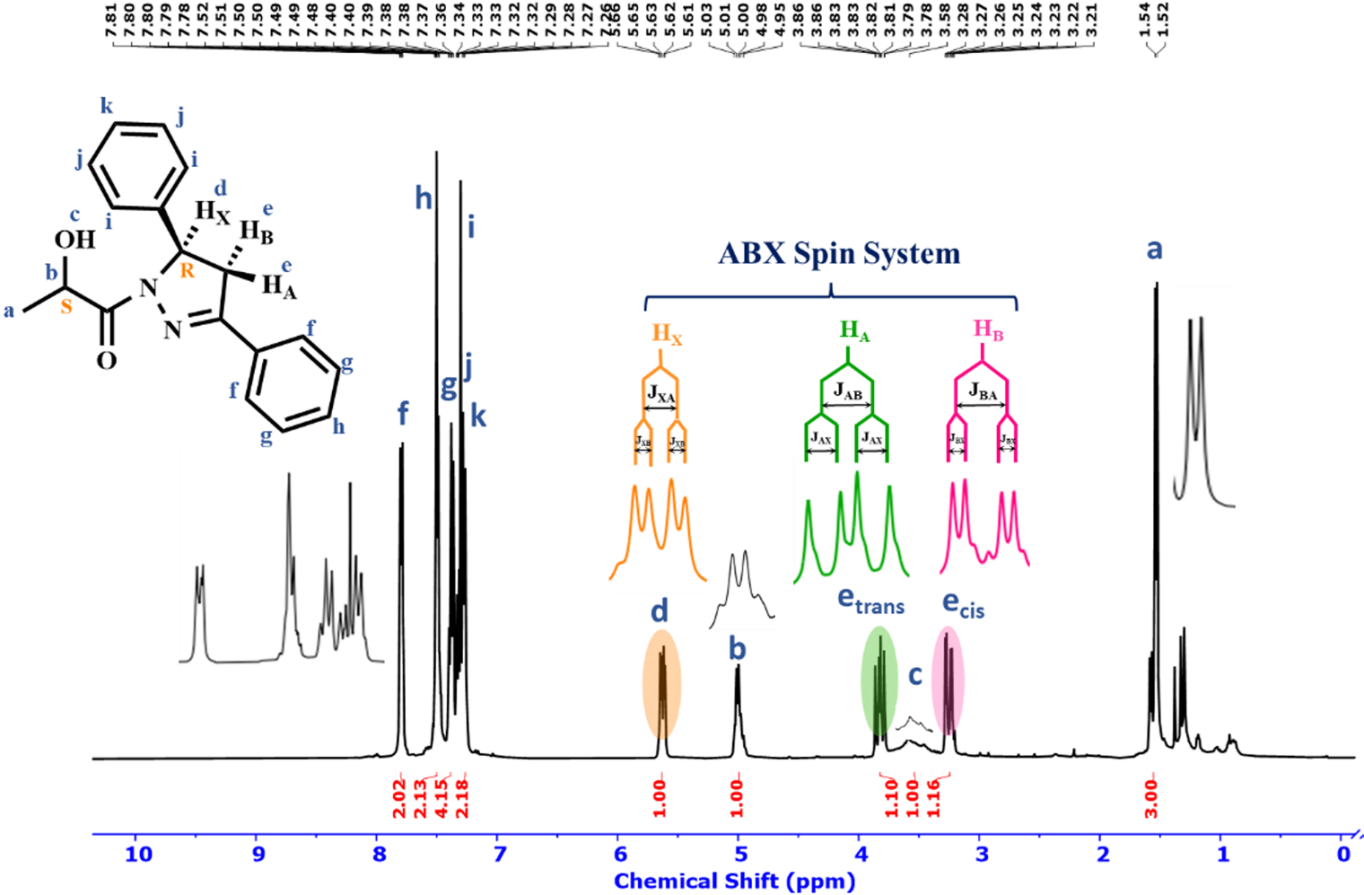
^1^H NMR (CDCl_3_, 400 MHz) spectrum of **7a**. Reaction condition: (*S*)-lactic hydrazide **3** (1 mmol), Chalcone **6a** (1 mmol), EtOH (10 ml), NaOH (0.5 mmol), Reflux, 24 h.

In the IR spectra of **7a**, absorption bands at 3424 cm^− 1^ indicated the presence of OH.

stretching on the chiral center. Absorption bands in the 3061, 2924 cm^− 1^ regions are related to Ar-H and C-H sp^3^ stretching, respectively. Absorption bands at 1641, 1595, 1442, 1125, and 1041 are related with C = O, C = N, C = C, C-N, and C-O, respectively (Fig. S1, Sup.data). The ^1^H NMR spectra of **7a** displayed three characteristic doublet of doublets corresponding to the stereogenic center proton (5-H pyrazoline) and the diastereotopic methylene protons of the pyrazoline ring (4-H_trans_ pyrazoline) and (4-H_cis_ pyrazoline) (ABX spin system) at *δ* 5.63, 3.82, 3.24 ppm for H_x_, H_A_ and H_B_ respectively. These results are consistent with the ^1^H NMR data reported in the literature^36^, which is an indication of pyrazoline ring formation (Fig. 1). The ^13^C NMR **7a** data also confirmed the results. The chemical shifts of imine, methine, and methylene carbons of the pyrazoline ring were found at 155, 66, and 42 ppm, respectively (Fig. S3, Sup.data).

These results encouraged us to synthesize a new series of model reaction product analogs. The reaction of **3** and **6(a-l)** gave title molecules **7(a-l)** under model reaction conditions, and the results are cited in Table 1. The structure of all products was studied and elucidated by IR, ^1^H, ^13^C NMR, and Mass spectroscopic data.

**Table 1:** Diastereoselective synthesis of Lacticpyrazolines^[a]^.


Entry	R_1_	R_2_	R_3_	R_4_	Chalcone 6(a-l)	Product 7(a-l)	Yield [%]^b^
1	H	H	H	H	6a	7a	57
2	H	H	H	Cl	6b	7b	63
3	H	H	Cl	Cl	6c	7c	59
4	H	H	H	CH_3_	6d	7d	34
5	H	OH	H	H	6e	7e	24
6	H	OH	H	Cl	6f	7f	28
7	H	OH	Cl	Cl	6 g	7 g	27
8	H	OH	H	CH_3_	6 h	7 h	25
9	CH_3_	H	H	H	6i	7i	33
10	CH_3_	H	H	Cl	6j	7j	34
11	CH_3_	H	H	Cl	6k	7k	36
12	CH_3_	H	H	CH_3_	6 L	7 L	31

^[a]^ (*S*)-lactichydrazide (1 mmol), chalcone (1 mmol), NaOH (0.5 mmol or 50 mol%), under reflux conditions in 10 ml EtOH, at 80 °C.

^[b]^ Isolated yield.

**Fig. 2:**
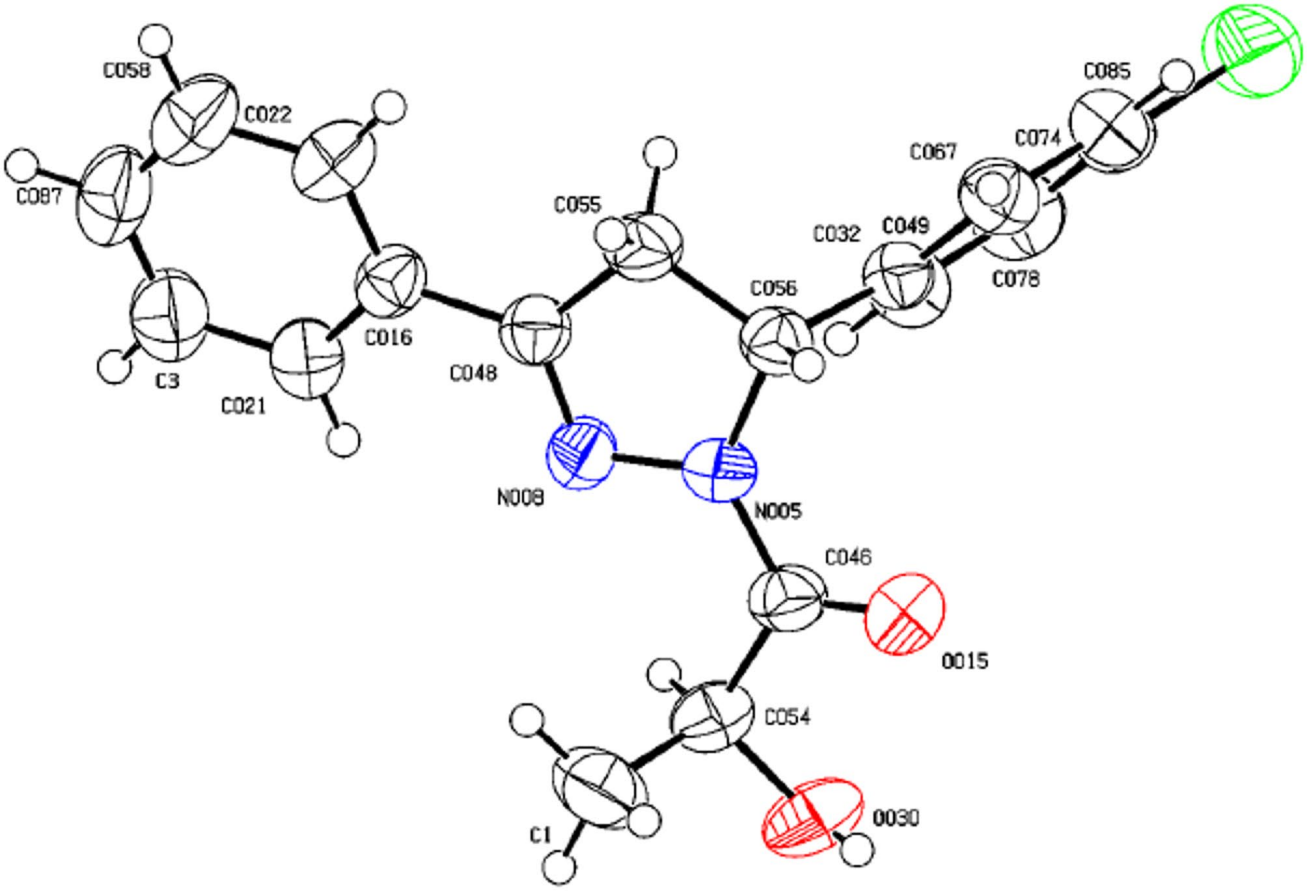
ORTEP representation of compound **7b** (CCDC: 2457374). Reaction condition: (*S*)-lactic hydrazide **3** (1 mmol), Chalcone **6b** (1 mmol), EtOH (10 ml), NaOH (0.5 mmol), Reflux, 24 h.

Unfortunately, among the obtained products, only **7b** can be crystallized as a single crystal. The other compounds produced were obtained as amorphous solids, which precluded their diffractometric analysis. For the amorphous solid products, the absolute configuration of the newly formed stereocenter in the pyrazoline ring can only be tentatively assigned by analogy with **7b**. In the case of **7b**, X-ray diffractometric analysis elucidated unequivocally the absolute configuration of the newly formed stereocenter in the 5-position of the pyrazoline ring as (*R*) configuration (Fig. 2).

The compound crystallizes in the orthorhombic crystal system with space group P2₁2₁2₁. The unit-cell parameters are a = 5.2285(10) Å, b = 17.006(3) Å, c = 18.560(4) Å, with a cell volume of 1650.3(6) Å³ and Z = 4. The refinement converged satisfactorily (R1 = 0.0550, wR2 = 0.1305), supporting a reliable structural model. The ORTEP plot clearly shows the (*S*,*R*)-relationship between the stereocenters of the pyrazoline ring, and the absolute configuration is consistent with nucleophilic attack from the Si-face as proposed in the mechanistic pathway. Crystallographic data for **7b** have been deposited at the Cambridge Crystallographic Data Centre (CCDC: 2457374). Additional crystallographic tables and refinement details are provided in the Supporting Information.

The X-ray result, TLC test, and ^1^H NMR spectra of crude reaction mixture showed that the reactions proceeded with approximately 100% diastereoselectivity giving exclusively the corresponding diastereoisomers that have (*R*)-configuration of the chiral carbon in their pyrazoline ring moiety.

**Scheme 2:**
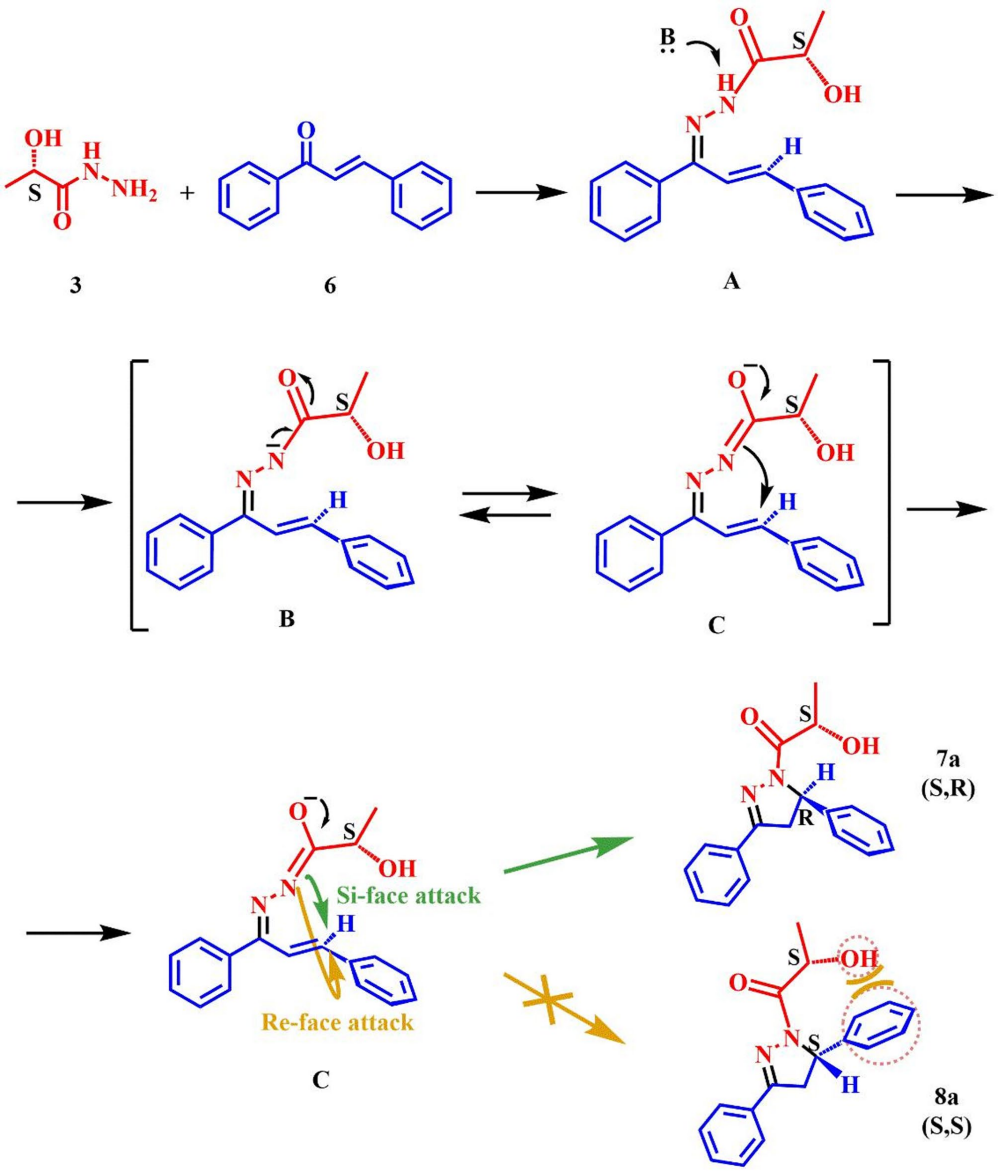
Proposed mechanism of lacticpyrazoline synthesis. B: NaOH, NaOEt.

A proposed mechanism for the formation of lactic pyrazolines is presented in Scheme 2. Initially, the nucleophilic attack of lactic hydrazide on the chalcone carbonyl forms intermediate (A) Under basic conditions, the amide nitrogen is deprotonated, forming intermediate (B) Intermediate B interacts with intermediate C through electron delocalization. In intermediate C, an intramolecular Aza-Michael addition takes place. The resonance delocalization of the negative charge from the oxygen toward the imine double bond in intermediate C facilitates this step. As a result, a nucleophilic attack on the Michael-acceptor site of the chalcone leading to intramolecular cyclization from the Si faces. We believe that steric hindrance between the hydroxyl group of lactic hydrazide and the chalcone phenyl ring prevents intramolecular cyclization from the Re face of the C intermediate (Scheme 2). Therefore, (*S*,*R*)-diastereomer is obtained as the only product of the reaction.

In the other study, we performed the DFT calculations on all double (*S*,*R*)-pyrazoline and (*S*,*S*)-pyrazoline derivatives (Table 2). The calculation method was as follows:

The structures of (*S*,*R*) and (*S*,*S*) diastereomers of the synthesized molecules were optimized by the B97 functional, including long-range correction by taking empirical dispersion into account as wB97X-D^37,38^. For all of the atoms 6-311 + G(d, p) basis set with diffuse and polarization functions was applied^39–42^. All of the calculations were performed by the GAMESS quantum chemistry program^42^. Then, the relative instabilities of the diastereomers obtained by the density functional theory (DFT) method are given in Table 2. It is noticeable that the relative instability for the related diastereomers is obtained by taking the electronic energy of the more stable isomer as zero and the difference between the electronic energies of the more stable isomer and the less stable one as the relative instability for the less stable isomer. The results of this study indicated that among all double diastereomers, the (*S*,*R*)-diastereomer is more stable than the (*S*,*S*)-diastereomer, and therefore, has a high probability of formation.

**Table 2:** The relative instability (in kJ/mol) for diastereomers of synthesized molecules obtained by DFT calculations.

Molecule	(*S*,*R*)	(*S*,*S*)	Molecule	(*S*,*R*)	(*S*,*S*)
7a	0.00	9.40	7 g	0.00	27.18
7b	0.00	10.26	7 h	0.00	8.76
7c	0.00	27.14	7i	0.00	9.38
7d	0.00	8.88	7j	0.00	10.20
7e	0.00	9.22	7k	0.00	27.30
7f	0.00	10.33	7 L	0.00	8.09

The antibacterial effects of the synthesized compounds are shown in Table 3. The well-diffusion method was performed using 50 µg of the synthesized compounds, and the results were reported as inhibitory zone diameter (IZD). Also, in the agar dilution method, the antibacterial effects were determined by reporting the minimum inhibitory concentration (MIC) values using different concentrations of the synthesized compounds from 4 to 512 µg/ml in DMSO.

The majority of the compounds had either no or weak antibacterial activity against both Gram-positive and Gram-negative bacteria. However, compounds **7g** and **7j** had a considerable effect against Gram-positive S. aureus. In addition, compound **7h** demonstrated activity against P. aeruginosa and A. baumannii. Although the antibacterial activity of components **7g**,** 7h** and **7j** were lower than ciprofloxacin, but these levels of activity show that these components can be selected for further analysis and production of more powerful antimicrobial compounds.

According to previous reports, different pyrazoline derivatives have exhibited a wide range of antibacterial activities. In one study, pyrazoline–acylthiourea hybrids bearing electron-withdrawing groups showed strong antibacterial activity with low MIC values^43^. Another study on pyrazoline and hydrazone derivatives reported weak-to-moderate activity (MIC 32–512 µg/mL), which is consistent with the results obtained in our work^44^. Furthermore, chalcone-derived pyrazolines demonstrated moderate to relatively good activity depending on their substituents, while other derivatives showed limited activity^45^.

**Table 3:** Antibacterial activity of synthesized components against 4 bacterial standard species.

Entry	Products	*S. aureus*		*P. aeruginosa*		*A. baumannii*		*K. pneumoniae*	
		IZD^[a]^	MIC^[b]^	IZD	MIC	IZD	MIC	IZD	MIC
1	7a	< 8	> 512	< 8	> 512	< 8	> 512	< 8	> 512
2	7b	< 8	> 512	< 8	> 512	< 8	> 512	< 8	> 512
3	7c	< 8	> 512	< 8	> 512	< 8	> 512	< 8	> 512
4	7d	< 8	> 512	< 8	> 512	< 8	> 512	< 8	> 512
5	7e	< 8	> 512	< 8	> 512	< 8	> 512	< 8	> 512
6	7f	< 8	> 512	< 8	> 512	< 8	> 512	< 8	> 512
7	7 g	12	264	< 8	> 512	< 8	> 512	< 8	> 512
8	7 h	< 8	> 512	10	512	10	512	< 8	> 512
9	7i	< 8	> 512	< 8	> 512	< 8	> 512	< 8	> 512
10	7j	14	264	< 8	> 512	< 8	> 512	< 8	> 512
11	7k	< 8	> 512	< 8	> 512	< 8	> 512	< 8	> 512
12	7 L	< 8	> 512	< 8	> 512	< 8	> 512	< 8	> 512
13	Cip^[c]^	25	< 1	24	1	25	< 1	25	< 1

^[a]^IZD: Inhibition Zone Diameter (mm), ^[b]^MIC: Minimum Inhibitory Concentration (µg/mL), ^[c]^Cip: Ciprofloxacin.

## Conclusion

In this work, we developed a simple and diastereoselective method to prepare pyrazoline derivatives using (*S*)-lactic hydrazide and different chalcones. Taking advantage of the natural chirality of the starting material, we obtained (*S*,*R*)-pyrazoline derivatives with good yields (24–63%) and excellent control over their stereochemistry. The structures of compounds **7(a–l)** were confirmed by spectroscopic analysis, while the absolute configuration of compound **7b** was determined by single-crystal X-ray diffraction (XRD). The observed diastereoselectivity was explained using a mechanistic approach based on intramolecular Aza-Michael addition, with the reaction proceeding with nearly 100% diastereoselectivity toward the formation of the (*S*,*R*)-diastereomer, predominantly from the Si-face. The antibacterial evaluation demonstrated that compounds **7g** (IZD: 12 mm, MIC: 264 µg/ml) and **7j** (IZD: 14 mm, MIC: 264 µg/ml) exhibited considerable activity against Gram-positive S. aureus. Additionally, compound **7h** (IZD: 10 mm, MIC: 512 µg/ml) showed moderate antibacterial effects against P. aeruginosa and A. baumannii. This diastereoselective protocol also provides a green and cost-effective synthetic approach, utilizing a naturally occurring bio-based precursor, proceeding under catalyst-free conditions, and using ethanol as an environmentally friendly solvent. and highlights the usefulness of chiral synthesis for accessing a variety of biologically active heterocyclic molecules.

## Experimental

### Chemicals and bacterial strains

The synthesis of (*S*)-lactic hydrazide was carried out following the published protocol^46^. Additionally, the chalcones were prepared using the procedure described in the paper^47^, and their structure was verified using NMR, X-ray, IR and Mass spectroscopy. The NaOH, solvents, and Silicagel were purchased from Fluka and Merck companies. All commercially available reagents and solvents were used without purification. Four bacterial strains, including one gram-positive bacterium (S. aureus ATCC 6538p) and three gram-negative bacteria (A. baumannii ATCC 19606, P. aeruginosa PO1, and K. pneumoniae ATCC 700603), were purchased from the Pasteur Institute of Iran.

### Characterization

FT-IR spectra were recorded on a Nicolet iS10 instrument. The compounds were visualized with UV light (254 nm). The NMR spectra were recorded on a Bruker 400 and 250 MHz spectrometer for ¹H NMR, and 101 and 63 MHz for ¹³C NMR, in CDCl₃. The solvents were extracted using a rotary evaporator at a lower pressure. The melting points were determined in open capillaries with a Gallenkamp melting point device mpd350.bm2.5 and are uncorrected. Optical rotation was measured with a Polarimeter model KRUSS P3000. Elemental analysis of C, H, and N was done by the CHN analyzer Perkin-Elmer, 2400 Series II. X-ray diffraction data were collected using a MARResearch 345 dtb imaging-plate detector. Mass spectra were recorded with an Agilent G708 1B MSD spectrometer operating at an ionization potential of 70 eV. Proton chemical shifts are reported relative to the residual proton signals of deuterated solvent CDCl_3_ (7.26 ppm) or TMS. Carbon chemical shifts were internally referenced to the deuterated solvent signals in CDCl_3_ (77.16 ppm). Chemical shifts are reported in *δ* (parts per million) values. Coupling constants (J) are reported in hertz. Proton coupling patterns were described as singlet (s), doublet (d), triplet (t), quartet (q), and multiplet (m). The antibacterial activity of the synthesized compounds was assessed by four bacterial species using the Agar Well diffusion (AWD) and Agar dilution methods.

### General procedure for the synthesis of pyrazoline derivatives 7(a − l)

(*S*)-Lactic hydrazide (1 mmol) and different substituted chalcones (1 mmol) in the presence of sodium hydroxide (0.5 mmol) in 10 ml of absolute ethanol were refluxed for 24 h. In the case of a series of products **(7b**, **7c**, **7k)**, after 24 h of reflux, the progress of the reaction was monitored by TLC (n-hexane: ethyl acetate = 2:4), and then the reaction mixture was cooled and poured onto crushed ice. The solid obtained was filtered and recrystallized^48^. About other products, after 24 h, the solid form product was not observed. Therefore, firstly, the solvents were moved by using a rotary evaporator at a lower pressure. The mixture was dissolved in ethyl acetate (5 ml), and the organic layer was washed with water (3 × 15 ml) and dried with anhydrous MgSO_4_. The reaction mixture was purified with chromatography plates using ethyl acetate∶n-hexane (2:4) as the eluent. It is noteworthy that in all of the reactions, the starting materials were not consumed completely, even with an increase in the reaction times to 48 h.

### Physical and spectroscopic data of isolated products

#### 1-(3,5-Diphenyl-4,5-dihydro-1*H*-pyrazol-1-yl)-2-hydroxypropan-1-one (7a)

Yellow solid, yield 57%; m.p: 108–110 °C; $\:{\left[{\upalpha\:}\right]}_{\mathrm{D}}^{20}\:$= 90 (c 0.002 in EtOAC); IR (KBr, cm^−1^): 3424 (OH), 3061 (Ar-H), 2924 (C-H), 1641 (C = O),1595 (C = N), 1442 (C = C), 1125 (C-N), 1041 (C-O); ^1^H NMR (400 MHz, CDCl_3_): *δ* (ppm) 7.78–8.81 (d, *J* = 8.0 Hz, 2 H, CH-Ar), 7.25–7.53 (m, 8 H, CH-Ar), 5.63 (dd, *J* = 12.0, 4.0 Hz, 1H, 5-H pyrazoline), 5.00 (q, *J* = 8.0 Hz, 1H, CH-O), 3.82 (dd, *J* = 16.0, 12.0 Hz, 1H, 4-H_trans_ pyrazoline), 3.58 (s, 1H, OH), 3.24 (dd, *J* = 16.0, 4.0 Hz, 1H, 4-H_cis_ pyrazoline), 1.53 (d, J= 8.0 Hz, 3 H, CH_3_); ^13^C NMR (101 MHz, CDCl_3_): *δ* (ppm) 172.8, 155.5, 141.0, 130.9, 129.1, 128.9, 128.0, 126.8, 125.5, 125.4, 66.6, 60.8, 42.0, 21.0. MS (EI): m/z (%) for C_18_H_18_N_2_O_2_ found 294.1 (M^+^, 42), 222.2 (100); calcd 294.35.

#### 1-(5-(4-Chlorophenyl)-3-phenyl-4,5-dihydro-1*H*-pyrazol-1-yl)-2-hydroxy propan-1-one (7b)

White solid, yield 63%; m.p: 193–195 °C ; $\:{\left[{\upalpha\:}\right]}_{\mathrm{D}}^{20}\:$= 250 (c 0.002 in EtOAC); IR (KBr, cm^−1^): 3416 (OH), 3029 (Ar-H), 2970 (C-H), 1651 (C = O), 1595 (C = N), 1490 (C = C), 1129 (C-N), 1036 (C-O); Elemental analysis Calcd for C_18_H_17_ClN_2_O_2_: C 65.75, H 5.21, N 8.52, found: C 63.51, H 5.03, N 8.15. ^1^H NMR (400 MHz, CDCl_3_): *δ* (ppm) 7.78–7.81 (d, *J* = 8.0 Hz, 2 H, CH-Ar), 7.47–7.54 (m, 3 H, CH-Ar),7.33–7.37 (d, *J* = 8.0 Hz, 2 H, CH-Ar), 7.20–7.23 (d, *J* = 8.0 Hz, 2 H, CH-Ar), 5.59 (dd, *J* = 12.0, 4.0 Hz, 1H, 5-H pyrazoline), 4.99 (q, *J* = 8.0 Hz, 1H, CH-O), 3.83 (dd, *J* = 16.0, 12.0 Hz, 1H, 4-H_trans_ pyrazoline), 3.50 (s, 1H, OH), 3.21 (dd, *J* = 16.0, 4.0 Hz, 1H, 4-H_cis_ pyrazoline), 1.52 (d, *J* = 8.0 Hz, 3 H, CH_3_). ^13^C NMR (101 MHz, CDCl_3_): *δ* (ppm) 172.9, 155.4, 139.5, 133.9, 131.0, 130.6, 129.3, 128.9, 127.1, 126.8, 66.6, 60.2, 41.9, 20.9. MS (EI): m/z (%) for C_18_H_17_ClN_2_O_2_ found 328.1 (M^+^, 1), 268.1 (100); calcd 328.80.

#### 1-(5-(2,4-Dichlorophenyl)-3-phenyl-4,5-dihydro-1*H*-pyrazol-1-yl)-2-hydroxy propan-1-one (7c)

White solid, yield 59%; m.p: 204–206 °C ; $\:{\left[{\upalpha\:}\right]}_{\mathrm{D}}^{20}\:$= 815 (c 0.002 in EtOAC); IR (KBr, cm^−1^): 3462 (OH), 3082 (Ar-H), 2970 (C-H), 1648 (C = O), 1595 (C = N), 1473 (C = C), 1129 (C-N), 1040(C-O); ^1^H NMR (400 MHz, CDCl_3_): *δ* (ppm) 8.01 (d, *J* = 8.0 Hz, 1H, CH-Ar), 7.77 (d, *J* = 8.0 Hz, 2 H, CH-Ar), 7.48 (m, 3 H, CH-Ar), 7.25 (dd, *J* = 8.0, 4.0 Hz, 2 H, CH-Ar), 7.04 (d, *J* = 8.0 Hz, 1H), 5.92 (dd, *J* = 12.0, 4.0 Hz, 1H, 5-H pyrazoline), 5.06 (quintet, *J* = 8.0 Hz, 1H, CH-O), 3.91 (dd, *J* = 16.0, 12.0 Hz, 1H, 4-H_trans_ pyrazoline), 3.50 (s, 1H, OH), 3.12 (dd, *J* = 16.0, 4.0 Hz, 1H, 4-H_cis_ pyrazoline), 1.54 (d, *J* = 8.0 Hz, 3 H, CH_3_); ^13^C NMR (101 MHz, CDCl_3_): *δ* (ppm) 172.1, 155.7, 136.4, 134.3, 132.5, 131.1, 130.5, 129.9, 128.9, 128.1, 127.8, 126.8, 66.6, 57.9, 41.0, 20.9. MS (EI): m/z (%) for C_18_H_16_Cl_2_N_2_O_2_ found 363.1 (M^+^, 2), 79.9 (100); calcd 363.24.

####  2-Hydroxy-1-(3-phenyl-5-(p-tolyl)-4,5-dihydro-1 *H* -pyrazol-1-yl)propan-1-one (7d)

Yellow solid yield, 34%; m.p: 118–120 °C; $\:{\left[{\upalpha\:}\right]}_{\mathrm{D}}^{20}\:$= 100 (c 0.002 in EtOAC); IR (KBr, cm^-1^): 3432 (OH), 3032 (Ar-H), 2920 (C-H), 1651 (C = O), 1562 (C = N), 1433 (C = C), 1129 (C-N), 1040 (C-O); ^1^H NMR (400 MHz, CDCl_3_): *δ* (ppm) 7.78–8.81 (m, *J* = 8.0 Hz, 2 H, CH-Ar), 7.46–7.53 (m, 4 H, CH-Ar), 7.19 (s, 3 H, CH-Ar), 5.59 (dd, *J* = 12.0, 4.0 Hz, 1H, 5-H pyrazoline), 4.99 (q, *J* = 8.0 Hz, 1H, CH-O), 3.80 (dd, *J* = 16.0, 12.0 Hz, 1H, 4-H_trans_ pyrazoline), 3.57 (s, 1H, OH), 3.24 (dd, *J* = 16.0, 4.0 Hz, 1H, 4-H_cis_ pyrazoline), 2.36 (s, 3 H, CH_3_), 1.53 (d, *J* = 8.0 Hz, 3 H, CH_3_); ^13^C NMR (101 MHz, CDCl_3_): *δ* (ppm) 172.8, 155.5, 138.2, 137.8, 130.9, 130.8, 129.7, 128.9, 126.8, 125.5, 66.6, 60.7, 42.1, 21.1, 21.0. MS (EI): m/z (%) for C_19_H_20_N_2_O_2_ found 308.1 (M^+^, 26), 236.1 (100); calcd 308.38.

####  2-Hydroxy-1-(3-(2-hydroxyphenyl)-5-phenyl-4,5-dihydro-1*H* -pyrazol-1-yl) propan-1-one (7e)

Yellow solid, yield 24%; m.p: 169–171 °C; $\:{\left[{\upalpha\:}\right]}_{\mathrm{D}}^{20}\:$= 25 (c 0.002 in EtOAC); IR (KBr, cm^-1^): 3432 (OH), 2917 (C-H), 1644 (C = O), 1556 (C = N), 1410 (C = C), 1129 (C-N), 1030 (C-O); ^1^H NMR (400 MHz, CDCl_3_): *δ* (ppm) 10.05 (s, 1H, OH-Ph), 7.36–7.48 (m, 5 H, CH-Ar), 7.29–7.31 (d, *J* = 8.0 Hz, 2 H, CH-Ar), 7.14 (d, *J* = 8.0 Hz, 1 H, CH-Ar), 7.01 (t, *J* = 8.0 Hz, 1 H, CH-Ar), 5.62 (dd, *J* = 12.0, 4.0 Hz, 1H, 5-H pyrazoline), 4.93 (q, *J* = 8.0 Hz, 1H, CH-O), 3.97 (dd, *J* = 16.0, 12.0 Hz, 1H, 4-H_trans_ pyrazoline), 3.60 (s, 1H, OH), 3.41 (dd, *J* = 16.0, 4.0 Hz, 1H, 4-H_cis_ pyrazoline), 1.53 (d, *J* = 8.0 Hz, 3 H, CH_3_); ^13^C NMR (101 MHz, CDCl_3_): *δ* (ppm) 172.1, 158.0, 157.8, 132.9, 129.2, 128.7, 128.3, 125.6, 125.4, 119.9, 117.3, 114.7, 66.2, 59.3, 42.5, 20.9. MS (EI): m/z (%) for C_18_H_18_N_2_O_3_ found 310.2 (M^+^, 32), 238.2 (100); calcd 310.35.

#### 1-(5-(4-Chlorophenyl)-3-(2-hydroxyphenyl)-4,5-dihydro-1*H*-pyrazol-1-yl)-2-hydroxypropan − 1-one (7f)

Yellow solid, yield 28%; m.p: 206–208 °C, $\:{\left[{\upalpha\:}\right]}_{\mathrm{D}}^{20}\:$= 220 (c 0.002 in EtOAC); IR (KBr, cm^−1^): 3435 (OH), 3065 (Ar-H), 2926 (C-H), 1658 (C = O),1592 (C = N), 1416 (C = C), 1086 (C-N), 1010 (C-O); ^1^H NMR (400 MHz, CDCl_3_): *δ* (ppm) 10.24 (s, 1H, OH-Ph), 7.42 (t, *J* = 8.0 Hz, 1H, CH-Ar), 7.35 (d, *J* = 8.0 Hz, 2 H, CH-Ar), 7.26 (d, *J* = 8.0 Hz, 1 H, CH-Ar), 7.23 (d, *J* = 8.0 Hz, 1 H, CH-Ar), 6.98 (t, *J* = 8.0 Hz, 1H, CH-Ar), 5.57 (dd, *J* = 12.0, 4.0 Hz, 1H, 5-H pyrazoline), 4.73 (q, *J* = 8.0 Hz, 1H, CH-O), 3.91 (dd, *J* = 16.0, 12.0 Hz, 1H, 4-H_trans_ pyrazoline), 3.59 (s, 1H, OH), 3.32 (dd, *J* = 16.0, 4.0 Hz, 1H, 4-H_cis_ pyrazoline), 1.32 (d, *J* = 8.0 Hz, 3 H, CH_3_); ^13^C NMR (101 MHz, CDCl_3_): *δ* (ppm) 173.8, 160.8, 157.7, 132.5, 129.2, 128.4, 127.2, 124.0, 119.8, 117.2, 116.7, 115.0, 57.9, 42.6, 29.7, 22.1. MS (EI): m/z (%) for C_18_H_17_ClN_2_O_3_ found 344.1 (M^+^, 1), 63.9 (100); calcd 344.80.

####  1-(5-(2,4-Dichlorophenyl)-3-(2-hydroxyphenyl)-4,5-dihydro-1*H* -pyrazol-1-yl)-2-hydroxypropan-1-one (7 g)

Yellow solid, yield 27%; m.p: 120–124 °C, $\:{\left[{\upalpha\:}\right]}_{\mathrm{D}}^{20}\:$= 70 (c 0.002 in EtOAC); IR (KBr, cm^-1^): 3427 (OH), 3064 (Ar-H), 2924 (C-H), 1658 (C = O),1591 (C = N), 1468 (C = C), 1141 (C-N), 1038 (C-O); ^1^H NMR (250 MHz, CDCl_3_): *δ* (ppm) 9.89 (s, 1H, OH-Ph), 6.92–7.55 (m, 7 H, CH-Ar), 5.86 (dd, *J* = 12.5, 5.0 Hz, 1H, 5-H pyrazoline), 4.95 (q, *J* = 7.5 Hz, 1H, CH-O), 3.99 (dd, *J* = 20.0, 12.5 Hz, 1H, 4-H_trans_ pyrazoline), 3.53 (s, 1H, OH), 3.21 (dd, *J* = 20.0, 5.0 Hz, 1H, 4-H_cis_ pyrazoline), 1.27 (d, *J* = 7.5 Hz, 3 H, CH_3_); ^13^C NMR (63 MHz, CDCl_3_): *δ* (ppm) 172.3, 162.8, 158.2, 133.1, 132.5, 130.1, 128.7, 127.8, 127.0, 124.5, 123.9, 119.9, 119.1, 117.3, 114.4, 66.2, 56.5, 41.5, 20.8. MS (EI): m/z (%) for C_18_H_16_Cl_2_N_2_O_3_ found 379.1 (M^+^, 4), 161.1 (100); calcd 379.24.

####  2-Hydroxy-1-(3-(2-hydroxyphenyl)-5-(p-tolyl)-4,5-dihydro-1*H* -pyrazol-1-yl)propan-1-one (7 h)

Yellow solid, yield 25.14%; m.p: 169–171 °C; $\:{\left[\boldsymbol{\upalpha\:}\right]}_{\mathbf{D}}^{20}\:$= 10 (c 0.002 in EtOAC); IR (KBr, cm^-1^): 3430 (OH), 3056 (Ar-H), 2920 (C-H), 1655 (C = O), 1618 (C = N), 1439 (C = C), 1124 (C-N), 1038 (C-O); ^1^H NMR (400 MHz, CDCl_3_): *δ* (ppm) 10.04 (s, 1H, OH-Ph), 7.43 (t, *J* = 8.0 Hz, 1H, CH-Ar), 7.28 (d, *J* = 8.0 Hz, 1H, CH-Ar), 7.18 (d, *J* = 8.0 Hz, 4 H, CH-Ar), 7.12 (d, *J* = 8.0 Hz, 1 H, CH-Ar), 6.99 (t, *J* = 8.0 Hz, 1H, CH-Ar), 5.57 (dd, *J* = 12.0, 4.0 Hz, 1H, 5-H pyrazoline), 4.89 (q, *J* = 8.0 Hz, 1H, CH-O), 3.92 (dd, *J* = 16.0, 12.0 Hz, 1H, 4-H_trans_ pyrazoline), 3.57 (s, 1H, OH), 3.37 (dd, *J* = 16.0, 4.0 Hz, 1H, 4-H_cis_ pyrazoline), 1.52 (d, *J* = 8.0 Hz, 3 H, CH_3_); ^13^C NMR (101 MHz, CDCl_3_): *δ* (ppm) 172.1, 166.7, 157.8, 138.2, 137.5, 132.9, 129.9, 128.7, 125.6, 120.1, 117.3, 114.5, 66.2, 59.2, 42.5, 29.7, 21.2, 20.9. MS (EI): m/z (%) for C_19_H_20_N_2_O_3_ found 324.1 (M^+^, 33), 252.1 (100); calcd 324.38.

####  2-Hydroxy-1-(5-phenyl-3-(p-tolyl)-4,5-dihydro-1*H* -pyrazol-1-yl)propan-1-one (7i)

Yellow solid, yield 33%; m.p: 134–136 °C;$\:{\left[{\upalpha\:}\right]}_{\mathrm{D}}^{20}\:$= 90 (c 0.002 in EtOAC); IR (KBr, cm^-1^): 3440 (OH), 2921 (C-H), 1651 (C = O), 1604 (C = N), 1442 (C = C), 1125 (C-N), 1038 (C-O); ^1^H NMR (250 MHz, CDCl_3_): *δ* (ppm) 7.65 (d, *J* = 7.5 Hz, 2 H), 7.22–7.37 (m, 7 H), 5.57 (dd, *J* = 12.5 Hz, 5.0 Hz, 1H, 5-H pyrazoline), 4.96 (q, *J* = 7.5 Hz, 4 H), 3.76 (dd, *J* = 17.5, 12.5 Hz, 1H, 4-H_trans_ pyrazoline), 3.55 (s, 1H, OH), 3.19 (dd, *J* = 17.5, 5.0 Hz, 1H, 4-H_cis_ pyrazoline), 2.41 (s, 3 H, CH_3_-Ph), 1.49 (d, *J* = 7.5 Hz, 3 H, CH_3_); ^13^C NMR (63 MHz, CDCl_3_): *δ* (ppm) 172.6, 155.5, 141.3, 141.1, 138.1, 129.5, 129.0, 126.8, 125.5, 66.6, 60.6, 60.3, 42.0, 21.5, 20.9. MS (EI): m/z (%) for C_19_H_20_N_2_O_2_ found 308.2 (M^+^, 57), 236.2 (100); calcd 308.38.

#### 1-(5-(4-Chlorophenyl)-3-(p-tolyl)-4,5-dihydro-1*H*-pyrazol-1-yl)-2-hydroxy propan-1-one (7j)

Yellow solid, yield 34%; m.p: 262–264 °C; $\:{\left[{\upalpha\:}\right]}_{\mathrm{D}}^{20}$ = 200 (c 0.002 in EtOAC); IR (KBr, cm^−1^): 3438 (OH), 2925 (C-H), 1650(C = O),1573 (C = N), 1412 (C = C), 1130 (C-N), 1037 (C-O); ^1^H NMR (400 MHz, CDCl_3_): *δ* (ppm) 7.67 (d, *J* = 8.0 Hz, 2 H), 7.34 (d, *J* = 8.0 Hz, 2 H), 7.29 (d, *J* = 8.0 Hz, 2 H), 7.21 (d, *J* = 8.0 Hz, 2 H), 5.57 (dd, *J* = 12.0, 4.0 Hz, 1H, 5-H pyrazoline), 4.97 (q, *J* = 8.0 Hz, 1H), 3.80 (dd, *J* = 16.0, 12.0 Hz, 1H, 4-H_trans_ pyrazoline), 3.58 (s, 1H, OH), 3.19 (dd, *J* = 16.0, 4.0 Hz, 1H, 4-H_cis_ pyrazoline), 2.45 (s, 3 H, CH_3_-Ph), 1.51 (d, *J* = 8.0 Hz, 3 H, ); ^13^C NMR (101 MHz, CDCl_3_): *δ* (ppm) 172.7, 155.6, 141.5, 139.6, 133.9, 129.6, 129.3, 127.8, 127.1, 126.8, 66.6, 60.1, 41.9, 21.6, 20.9. MS (EI): m/z (%) for C_19_H_19_ClN_2_O_2_ found 342.1 (M^+^, 35), 270.1 (100); calcd 342.82.

#### 1-(5-(2,4-Dichlorophenyl)-3-(p-tolyl)-4,5-dihydro-1*H*-pyrazol-1-yl)-2-hydroxy propan-1-one (7k)

Yellow solid, yield 36%; m.p: 221–224 °C; $\:{\left[{\upalpha\:}\right]}_{\mathrm{D}}^{20}$ = 205 (c 0.002 in EtOAC); IR (KBr, cm^−1^): 3372 (OH), 3031 (Ar-H), 2921 (C-H), 1661 (C = O), 1584 (C = N), 1464 (C = C), 1140 (C-N), 1046 (C-O); ^1^H NMR (400 MHz, CDCl_3_): *δ* (ppm) 7.68 (d, *J* = 8.0 Hz, 2 H), 7.47 (s, 1H), 7.28 (d, *J* = 8.0 Hz, 2 H), 7.24 (d, *J* = 8.0 Hz, 1H), 7.03 (d, *J* = 8.0 Hz, 1H), 5.90 (dd, *J* = 12.0, 4.0 Hz, 1H, 5-H pyrazoline), 5.05 (q, *J* = 8.0 Hz, 1H), 3.88 (dd, *J* = 16.0, 12.0 Hz, 4-H_trans_ pyrazoline), 3.56 (s, 1H, OH), 3.10 (dd, *J* = 16.0, 4.0 Hz, 1H, 4-H_cis_ pyrazoline), 2.44 (s, 3 H, CH_3_-Ph), 1.53 (d, *J* = 8.0 Hz, 3 H, CH_3_); ^13^C NMR (101 MHz, CDCl_3_): *δ* (ppm) 172.7, 155.8, 141.6, 136.5, 134.26, 132.5, 129.9, 129.6, 127.8, 127.7, 126.9, 126.8, 66.6, 57.8, 41.1, 21.6, 20.9. MS (EI): m/z (%) for C_19_H_18_Cl_2_N_2_O_2_ found 377.1 (M^+^, 5), 304.1 (100); calcd 377.27.

#### 1-(3,5-Di-p-tolyl-4,5-dihydro-1*H*-pyrazol-1-yl)-2-hydroxypropan-1-one (7l)

Yellow solid, yield 31%; m.p: 146–149 °C ; $\:{\left[{\upalpha\:}\right]}_{\mathrm{D}}^{20}$= 165 (c 0.002 in EtOAC); IR (KBr, cm^−1^): 3431 (OH), 3030 (Ar-H), 2924 (C-H), 1651 (C = O), 1551 (C = N), 1438 (C = C), 1128 (C-N), 1041 (C-O); ^1^H NMR (400 MHz, CDCl_3_): *δ* (ppm) 7.68 (d, *J* = 8.0 Hz, 2 H), 7.29 (d, *J* = 8.0 Hz, 2 H), 7.21–7.12 (m, 4 H), 5.58 (dd, *J* = 12.0, 4.0 Hz, 1H, 5-H pyrazoline), 5.00 (q, *J* = 8.0 Hz, 1H), 4.81 (s, 1H), 3.78 (dd, *J* = 16.0, 12.0 Hz, 1H, 4-H_trans_ pyrazoline), 3.22 (dd, *J* = 16.0, 4.0 Hz, 1H, 4-H_cis_ pyrazoline), 2.45 (s, 3 H, CH_3_-Ph), 2.35 (s, 3 H, CH_3_-Ph), 1.53 (d, *J* = 8.0 Hz, 3 H, CH_3_); ^13^C NMR (101 MHz, CDCl_3_): *δ* (ppm) 172.7, 155.7, 141.3, 138.2, 137.7, 129.7, 129.6, 128.1, 126.8, 125.5, 66.6, 60.6, 42.1, 21.6, 21.2, 21.0. MS (EI): m/z (%) for C_20_H_22_N_2_O_2_ 322.2 (M^+^, 31), 91.1 (100); calcd 322.41.

## Supplementary Information

Below is the link to the electronic supplementary material.

Supplementary Material 1

## Data Availability

All data generated or analyzed during this study are included in this published article (and its Supplementary Information files) from the corresponding author on reasonable request.

## References

[1] Hu, S., Zhang, S., Hu, Y., Tao, Q. & Wu, A. A new selective pyrazoline-based fluorescent chemosensor for Cu2 + in aqueous solution. *Dyes Pigm.* **96** (2), 509–515 (2013).

[2] Zhang, T. T. et al. B.-X. A simple pyrazoline-based fluorescent probe for Zn2 + in aqueous solution and imaging in living neuron cells. *Sens. Actuators B*. **186**, 755–760 (2013).

[3] Wagner, A., Schellhammer, C. W. & Petersen, S. Aryl-∆2‐pyrazolines as optical brighteners. *Angewandte Chemie Int. Ed. Engl.* **5** (8), 699–704 (1966).

[4] Kumar, C. K. et al. Ferrocenyl pyrazoline based multichannel receptors for a simple and highly selective recognition of Hg2 + and Cu2 + ions. *J. Organomet. Chem.* **780**, 20–29 (2015).

[5] Shi, H. B., Ji, S. J. & Bian, B. Studies on transition metal ions recognition properties of 1-(2-benzothiazole)-3-(2-thiophene)-2-pyrazoline derivatives. *Dyes Pigm.* **73** (3), 394–396 (2007).

[6] Wang, P. et al. 3-(2-Pyridyl)-2-pyrazoline derivatives: novel fluorescent probes for Zn2 + ion. *Tetrahedron Lett.* **42** (52), 9199–9201 (2001).

[7] Cyprych, K. et al. Spontaneous crystalization and aggregation of DCNP pyrazoline-based organic dye as a way to tailor random lasers. *J. Phys. D*. **48** (19), 195101 (2015).

[8] Vandana, T., Ramkumar, V. & Kannan, P. Synthesis and fluorescent properties of Poly (arylpyrazoline)’s for organic-electronics. *Opt. Mater.* **58**, 514–523 (2016).

[9] Arbačiauskienė, E. et al. Multifunctional polyconjugated molecules with Carbazolyl and Pyrazolyl moieties for optoelectronic applications. *Synth. Met.* **160** (5), 490–498 (2010).

[10] Jin, M. et al. Synthesis and properties of photoluminescence and electroluminescence of pyrazoline derivatives. *Synth. Met.* **140** (1), 37–41 (2004).

[11] Cherpak, V. et al. Properties of 2,6-di-tert.-butyl-4-(2,5-diphenyl-3,4-dihydro-2H-pyrazol-3-yl)-phenol as hole-transport material for life extension of organic light emitting diodes. *Opt. Mater.* **33** (11), 1727–1731 (2011).

[12] Anita; Chouhan, L. K., Ameta, S., Sharma, V. & Ranawat, P. S. Synthesis of pyrazoline derivatives and their Pharmacological activities: A review of the last decade. *ChemistrySelect* **9** (9), e202304483 (2024).

[13] Gabr, B. S., Shalabi, A. R., Said, M. F. & George, R. F. 3, 5-Disubstituted pyrazoline as a promising core for anticancer agents: mechanisms of action and therapeutic potentials. *Future Med. Chem.* **17** (6), 725–745 (2025). 40079157 10.1080/17568919.2025.2476393 PMC11938987

[14] Qadir, K. M. et al. Synthesis, molecular Docking study and antibacterial activity of new pyrazoline derivatives. *Bull. Chem. Soc. Ethiop.* **39** (5), 955–966 (2025).

[15] Yadav, C. S. et al. Synthesis, characterization and bio-evaluation of novel series of pyrazoline derivatives as potential antifungal agents. *Sci. Rep.* **15** (1), 1–20 (2025). 40295598 10.1038/s41598-025-98645-1 PMC12037995

[16] Al-Masoudi, W. A., Al-Masoudi, N. A., Saeed, B. A., Winter, R. & Pannecouque, C. Synthesis, in vitro anti-HIV activity, cytotoxicity, and computational studies of some new steroids and their pyrazoline and oxime analogues. *Russ. J. Bioorg. Chem.* **46**, 822–836 (2020).

[17] Zala, M., Vora, J. J., Khedkar, V. M. & Synthesis Characterization, antitubercular activity, and molecular Docking studies of pyrazolylpyrazoline-clubbed Triazole and tetrazole hybrids. *ACS Omega*. **8** (23), 20262–20271 (2023). 37323386 10.1021/acsomega.2c07267 PMC10268283

[18] Barghady, N. et al. Synthesis, characterization, mechanistic study, in-vitro and in-silico evaluation of antibacterial and antioxidant activities of novel pyrazole-pyrazoline hybrid systems. *J. Mol. Struct.* **1309**, 138087 (2024).

[19] Ravindar, L., Hasbullah, S. A., Rakesh, K. & Hassan, N. I. Pyrazole and pyrazoline derivatives as antimalarial agents: A key review. *Eur. J. Pharm. Sci.* **183**, 106365 (2023). 36563914 10.1016/j.ejps.2022.106365

[20] Havrylyuk, D., Zimenkovsky, B., Vasylenko, O. & Lesyk, R. Synthesis and anticancer and antiviral activities of new 2-Pyrazoline‐Substituted 4‐Thiazolidinones. *J. Heterocycl. Chem.* **50** (S1), E55–E62 (2013).

[21] Turkan, F., Cetin, A., Taslimi, P., Karaman, H. S. & Gulçin, İ. Synthesis, characterization, molecular Docking and biological activities of novel pyrazoline derivatives. *Arch. Pharm.* **352** (6), 1800359 (2019). 10.1002/ardp.201800359 31125504

[22] Sid, A. et al. 1-Formyl-3-phenyl-5-(4-isopropylphenyl)-2-pyrazoline: Synthesis, characterization, antimicrobial activity and DFT studies. *J. Mol. Struct.* **1121**, 46–53 (2016).

[23] Beyhan, N., Kocyigit-Kaymakcioglu, B., Gümrü, S. & Aricioglu, F. Synthesis and anticonvulsant activity of some 2-pyrazolines derived from chalcones. *Arab. J. Chem.* **10** S2073-S2081 (2017).

[24] Chen, M. et al. Copper-catalyzed diamination of alkenes of unsaturated ketohydrazones with amines. *Org. Lett.* **20** (3), 510–513 (2018). 29355325 10.1021/acs.orglett.7b03401

[25] Lellek, V. et al. An efficient synthesis of substituted pyrazoles from one-pot reaction of ketones, aldehydes, and hydrazine monohydrochloride. *Synlett* **29** (08), 1071–1075 (2018).

[26] Li, Y., Zhang, X., Lu, T. & Miao, Z. A regioselective synthesis of substituted pyrazolines via a cascade annulation of Huisgen Zwitterion with α-Cyano‐α, β‐unsaturated ketones under Solvent‐free heating conditions. *ChemistrySelect* **4** (35), 10352–10356 (2019).

[27] Babinski, D. J., Aguilar, H. R., Still, R. & Frantz, D. E. Synthesis of substituted pyrazoles via tandem cross-coupling/electrocyclization of enol triflates and diazoacetates. *J. Org. Chem.* **76** (15), 5915–5923 (2011). 21682322 10.1021/jo201042c PMC3155889

[28] Wang, G. et al. Asymmetric catalytic 1, 3-dipolar cycloaddition reaction of nitrile Imines for the synthesis of chiral spiro-pyrazoline-oxindoles. *Org. Lett.* **15** (1), 76–79 (2013). 23228061 10.1021/ol303097j

[29] Huang, H. et al. A one-step approach to N-(hetero) Aryl-3, 5-dinitropyrazoles from (hetero) Aryl amines. *Org. Lett.* **22** (15), 5866–5869 (2020). 32672468 10.1021/acs.orglett.0c01960

[30] Lin, L. & Feng, X. Catalytic strategies for diastereodivergent synthesis. *Chemistry–A Eur. J.* **23** (27), 6464–6482 (2017). 10.1002/chem.201604617 27859806

[31] Safaei, S. et al. Diastereoselective synthesis of pyrazolines using a bifunctional Brønsted acidic ionic liquid under Solvent‐Free conditions. *Adv. Synth. Catal.* **354** (16), 3095–3104 (2012).

[32] Zeng, Y. et al. Diastereodivergent synthesis of pyrazoline derivatives through [3 + 2] cycloaddition of Baylis–Hillman adducts and nitrilimines. *J. Heterocycl. Chem.* **55** (12), 2781–2791 (2018).

[33] Bucci, R., Clerici, F., Pellegrino, S. & Erba, E. Diastereoselective synthesis of pyrazolines by metal-free rearrangement of bicyclic triazolines. *Synthesis* **52** (19), 2892–2898 (2020).

[34] Du, Y., Liu, Y., Guo, H., Liu, R. & Zhou, R. Chemo-and diastereoselective synthesis of spirooxindole-pyrazolines and pyrazolones via P (NMe2) 3-mediated substrate-controlled annulations of azoalkenes with α-dicarbonyl compounds. *Org. Lett.* **25** (26), 4776–4781 (2023). 37358479 10.1021/acs.orglett.3c01348

[35] Qiu, P. W. et al. Synthesis of pyrazoline-derived N-vinyl nitrones through an unexpected selective [3 + 2] cycloaddition. *Org. Chem. Front.* **12** (11), 3403–3408 (2025).

[36] Kankanala, K. et al. Nonsteroidal Anti-inflammatory Drug‐based N‐Allylidene benzohydrazides and 1‐Acyl‐2‐pyrazolines: their synthesis as potential cytotoxic agents in vitro. *J. Heterocycl. Chem.* **52** (1), 105–113 (2015).

[37] Chai, J. D. & Head-Gordon, M. Long-range corrected hybrid density functionals with damped atom–atom dispersion corrections. *Phys. Chem. Chem. Phys.* **10** (44), 6615–6620 (2008). 18989472 10.1039/b810189b

[38] Chai, J. D. & Head-Gordon, M. Systematic optimization of long-range corrected hybrid density functionals. *The J. Chem. Phys.* **128** (8), (2008). 10.1063/1.2834918 18315032

[39] McLean, A. & Chandler, G. Contracted Gaussian basis sets for molecular calculations. I. Second row atoms, Z = 11–18. *J. Chem. Phys.* **72** (10), 5639–5648 (1980).

[40] Krishnan, R., Binkley, J. S., Seeger, R. & Pople, J. A. Self-consistent molecular orbital methods. XX. A basis set for correlated wave functions. *J. Chem. Phys.* **72** (1), 650–654 (1980).

[41] Clark, T., Chandrasekhar, J., Spitznagel, G. W. & Schleyer, P. V. R. Efficient diffuse function-augmented basis sets for anion calculations. III. The 3‐21 + G basis set for first‐row elements, Li–F. *J. Comput. Chem.* **4** (3), 294–301 (1983).

[42] Frisch, M. J., Pople, J. A. & Binkley, J. S. Self-consistent molecular orbital methods 25. Supplementary functions for Gaussian basis sets. *J. Chem. Phys.* **80** (7), 3265–3269 (1984).

[43] Saeed, A. et al. Novel pyrazoline linked acyl thiourea pharmacophores as antimicrobial, urease, amylase and α-glucosidase inhibitors: design, synthesis, SAR and molecular Docking studies. *RSC Adv.* **14** (2), 1018–1033 (2024). 38174269 10.1039/d3ra06812a PMC10759180

[44] AksÖz, B. E., GÜrpinar, S. S. & Eryilmaz, M. Antimicrobial activities of some pyrazoline and hydrazone derivatives. *Turkish J. Pharm. Sci.* **17** (5), 500 (2020). 10.4274/tjps.galenos.2019.42650 PMC7650741 33177930

[45] Tok, F., Doğan, M. O., Gürbüz, B. & Kaymakçıoğlu, B. Synthesis of novel pyrazoline derivatives and evaluation of their antimicrobial activity. *J. Res. Pharm.* **26** (5), 1453–1460 (2022).

[46] Noshiranzadeh, N., Heidari, A., Haghi, F., Bikas, R. & Lis, T. Chiral lactic hydrazone derivatives as potential bioactive antibacterial agents: Synthesis, spectroscopic, structural and molecular Docking studies. *J. Mol. Struct.* **1128**, 391–399 (2017).

[47] Suwito, H. et al. Design and synthesis of chalcone derivatives as inhibitors of the ferredoxin—Ferredoxin-NADP + reductase interaction of plasmodium falciparum: pursuing new antimalarial agents. *Molecules* **19** (12), 21473–21488 (2014). 25532844 10.3390/molecules191221473 PMC6271513

[48] Akhtar, M. J. et al. Synthesis of stable benzimidazole derivatives bearing pyrazole as anticancer and EGFR receptor inhibitors. *Bioorg. Chem.* **78**, 158–169 (2018). 29571113 10.1016/j.bioorg.2018.03.002

