# Supplementary material for: Diastereoselective synthesis of novel (S)-lactic pyrazoline derivatives and investigation of antibacterial capabilities
Source: Sci Rep. 2025 Dec 21;16:2999. doi: 10.1038/s41598-025-32881-3 (PMC12830598; doi:10.1038/s41598-025-32881-3)
Supplement: Supplementary file 1 — Supplementary Material 1 [file 41598_2025_32881_MOESM1_ESM.docx]

**Supporting Information**

**Diastereoselective Synthesis of Novel (*S*)-lactic Pyrazoline Derivatives and Investigation of Antibacterial Capabilities**

Maryam Gholami^1^, Gholamhassan Imanzadeh^*1^, Farhad Kabiri Esfahani^2^, Morteza Karami-Zarandi^3^, Amir Nasser Shamkhali^1^

^1^. Department of Chemistry, Faculty of Sciences, University of Mohaghegh Ardabili 56199-11367, Ardabil, Iran.

Email: Imanzad2000@yahoo.com; g_imanzadeh@uma.ac.ir

^2^. Department of Chemistry, Faculty of Sciences, University of Zanjan, Zanjan 45371-38791, Iran

^3^. Department of Microbiology, School of Medicine, Zanjan University of Medical Sciences, Zanjan, 4513956111, Iran

Corresponding author Email: [Imanzad2000@yahoo.com](mailto:Imanzad2000@yahoo.com); [g_imanzadeh@uma.ac.ir](mailto:g_imanzadeh@uma.ac.ir)

**Table of Contents**

[Experimental 4](#_Toc215400758)

[**Materials and instruments 4**](#_Toc215400759)

[**Synthesis of (*S*)-lactic hydrazide 3 5**](#_Toc215400760)

[**Synthesis of chalcone derivatives 6(a-l) 5**](#_Toc215400761)

[**General Procedure for the synthesis of pyrazoline derivatives 7(a−l) 5**](#_Toc215400762)

[IR, ^1^H NMR, ^13^C NMR spectrum analyses of products 6](#_Toc215400763)

[**Figure S1: FT-IR (KBr) spectrum of 7a 6**](#_Toc215400764)

[**Figure S2: ^1^H NMR (400 MHz, CDCl_3_) spectrum of 7a 6**](#_Toc215400765)

[**Figure S3:^13^C NMR (101 MHz, CDCl_3_) spectrum of 7a 7**](#_Toc215400766)

[**Figure S4. Mass spectrum of 7a 8**](#_Toc215400767)

[**Figure S5: FT-IR (KBr) spectrum of 7b 9**](#_Toc215400768)

[**Figure S6: ^1^H NMR (400 MHz, CDCl_3_) spectrum of 7b 9**](#_Toc215400769)

[**Figure S7: ^13^C NMR (101 MHz, CDCl_3_) spectrum of 7b 10**](#_Toc215400770)

[**Figure S8. Mass spectrum of 7b 11**](#_Toc215400771)

[**Figure S9: FT-IR (KBr) spectrum of 7c 12**](#_Toc215400772)

[**Figure S10: ^1^H NMR (400 MHz, CDCl_3_) spectrum of 7c 12**](#_Toc215400773)

[**Figure S11: ^13^C NMR (101 MHz, CDCl_3_) spectrum of 7c 13**](#_Toc215400774)

[**Figure S12. Mass spectrum of 7c 14**](#_Toc215400775)

[**Figure S13: FT-IR (KBr) spectrum of 7d 15**](#_Toc215400776)

[**Figure S14: ^1^H NMR (400 MHz, CDCl_3_) spectrum of 7d 15**](#_Toc215400777)

[**Figure S15: ^13^C NMR (101 MHz, CDCl_3_) spectrum of 7d 16**](#_Toc215400778)

[**Figure S16. Mass spectrum of 7d 17**](#_Toc215400779)

[**Figure S17: FT-IR (KBr) spectrum of 7e 18**](#_Toc215400780)

[**Figure S18: ^1^H NMR (400 MHz, CDCl_3_) spectrum of 7e 18**](#_Toc215400781)

[**Figure S19: 13C NMR (CDCl3, 101 MHz) spectrum of 7e 19**](#_Toc215400782)

[**Figure S20. Mass spectrum of 7e 20**](#_Toc215400783)

[**Figure S21: FT-IR (KBr) spectrum of 7f 21**](#_Toc215400784)

[**Figure S22: ^1^H NMR (400 MHz, CDCl3) spectrum of 7f 21**](#_Toc215400785)

[**Figure S23: ^13^C NMR (CDCl_3_, 101 MHz) spectrum of 7f 22**](#_Toc215400786)

[**Figure S24. Mass spectrum of 7f 23**](#_Toc215400787)

[**Figure S25: FT-IR (KBr) spectrum of 7g 24**](#_Toc215400788)

[**Figure S26: ^1^H NMR (250 MHz, CDCl3) spectrum of 7g 24**](#_Toc215400789)

[**Figure S27: ^13^C NMR (63 MHz, CDCl_3_) spectrum of 7g 25**](#_Toc215400790)

[**Figure S28. Mass spectrum of 7g 26**](#_Toc215400791)

[**Figure S29: FT-IR (KBr) spectrum of 7h 27**](#_Toc215400792)

[**Figure S30: ^1^H NMR (400 MHz, CDCl_3_) spectrum of 7h 27**](#_Toc215400793)

[**Figure S31: 13C NMR (101 MHz, CDCl3) spectrum of 7h 28**](#_Toc215400794)

[**Figure S32. Mass spectrum of 7h 29**](#_Toc215400795)

[**Figure S33: FT-IR (KBr) spectrum of 7i 30**](#_Toc215400796)

[**Figure S34: ^1^H NMR (250 MHz, CDCl_3_) spectrum of 7i 30**](#_Toc215400797)

[**Figure S35: ^13^C NMR (63 MHz, CDCl_3_) spectrum of 7i 31**](#_Toc215400798)

[**Figure S36. Mass spectrum of 7i 32**](#_Toc215400799)

[**Figure S37: FT-IR (KBr) spectrum of 7j 33**](#_Toc215400800)

[**Figure S38: ^1^H NMR (400 MHz, CDCl_3_) spectrum of 7j 33**](#_Toc215400801)

[**Figure S39: ^13^C NMR (101 MHz, CDCl_3_) spectrum of 7j 34**](#_Toc215400802)

[**Figure S40. Mass spectrum of 7j 35**](#_Toc215400803)

[**Figure S41: FT-IR (KBr) spectrum of 7k 36**](#_Toc215400804)

[**Figure S42: ^1^H NMR (400 MHz, CDCl_3_) spectrum of 7k 36**](#_Toc215400805)

[**Figure S43: ^13^C NMR (101 MHz, CDCl_3_) spectrum of 7k 37**](#_Toc215400806)

[**Figure S44. Mass spectrum of 7k 38**](#_Toc215400807)

[**Figure S45: FT-IR (KBr) spectrum of 7l 39**](#_Toc215400808)

[**Figure S46: ^1^H NMR (400 MHz, CDCl_3_) spectrum of 7l 39**](#_Toc215400809)

[**Figure S47: ^13^C NMR (101 MHz, CDCl_3_) 1spectrum of 7l 40**](#_Toc215400810)

[**Figure S48. Mass spectrum of 7l 41**](#_Toc215400811)

[Crystallographic data of compound 7b 41](#_Toc215400812)

[**Figure S49. ORTEP representation of compound 7b. 43**](#_Toc215400813)

[**Figure S50. Crystal structure of compound 7b 43**](#_Toc215400814)

# Experimental

## **Materials and instruments**

The synthesis of (*S*)-lactic hydrazide was carried out following the published protocol. ^1^ Additionally, the chalcones were prepared using the procedure described in the paper.^2^ and its structure was verified using NMR, X-ray, IR and Mass spectroscopy. The NaOH, solvents, and Silicalgel were purchased from Fluka and Merck companies. All commercially available reagents and solvents were used without purification. Chromatography plates were prepared using silica gel. Four bacterial strains, including one gram-positive bacterium (S. aureus ATCC 6538p) and three gram-negative bacteria (A. baumannii ATCC 19606, P. aeruginosa PO1, and K. pneumoniae ATCC 700603), were purchased from the Pasteur Institute of Iran.

FT-IR spectra were recorded on a Nicolet iS10 instrument. The compounds were visualized with UV light (254 nm). The NMR spectra were recorded on a Bruker 400 and 250 MHz spectrometer for ¹H NMR, and 101 and 63 MHz for ¹³C NMR, in CDCl₃. The solvents were extracted using a rotary evaporator at a lower pressure. The melting points were determined in open capillaries with a Gallenkamp melting point device mpd350.bm2.5 and are uncorrected. Optical rotation was measured with a Polarimeter model KRUSS P3000. Elemental analysis of C, H, and N was done by the CHN analyzer Perkin-Elmer, 2400 Series II. X-ray diffraction data were collected using a MARResearch 345 dtb imaging-plate detector. Mass spectra were recorded with an Agilent G708 1B MSD spectrometer operating at an ionization potential of 70 eV. Proton chemical shifts are reported relative to the residual proton signals of deuterated solvent CDCl3 (7.26 ppm) or TMS. Carbon chemical shifts were internally referenced to the deuterated solvent signals in CDCl3 (77.16 ppm). Chemical shifts are reported in δ (parts per million) values. Coupling constants (J) are reported in hertz. Proton coupling patterns were described as singlet (s), doublet (d), triplet (t), quartet (q), and multiplet (m). The antibacterial activity of the synthesized compounds was assessed by four bacterial species using the Agar Well diffusion (AWD) and Agar dilution methods.

##

## **Synthesis of (*S*)-lactic hydrazide 3**

(*S*)-Ethyl lactate and hydrazine hydrate 80% were mixed in equal amounts in the absence of any solvent and refluxed for 3 hours. The reaction mixture was obtained as an oily colorless liquid. This mixture was placed under pressure at room temperature for several days, and the final oily product was used in subsequent syntheses.^1^.

## **Synthesis of chalcone derivatives 6(a-l)**

A combination of acetophenone derivatives 4(a-c) (6 mmol) and benzaldehyde derivatives 5(a-d) (6 mmol) was mixed in 20 mL of ethanol, then 6 mL of 40% NaOH solution was added dropwise while keeping the temperature below 10°C. The mixture was stirred for 1 hour under these conditions, then stirred at room temperature for 4 hours. After that, the mixture was poured into ice-water; the solid that formed was filtered out and purified by recrystallization from aqueous ethanol. Thereafter, the reaction mixture was poured into ice water; the precipitated solid was filtered off and recrystallized from aqueous ethanol.^2^

## **General Procedure for the synthesis of pyrazoline derivatives 7(a−l)**

(*S*)-Lactic hydrazide (1 mmol) and different substituted chalcones (1 mmol) in the presence of sodium hydroxide (0.5 mmol) in 10 ml of absolute ethanol were refluxed for 24 h. In the case of a series of products **(7b, 7c, 7k)**, after 24 h of reflux, the progress of the reaction was monitored by TLC (n-hexane: ethyl acetate = 2:4), and then the reaction mixture was cooled and poured onto crushed ice. The solid obtained was filtered and recrystallized ^3^. About other products, after 24 h, the solid form product was not observed. Therefore, firstly, the solvents were moved by using a rotary evaporator at a lower pressure. The mixture was dissolved in ethyl acetate (5 ml), and the organic layer was washed with water (3×15 ml) and dried with anhydrous MgSO_4_. The reaction mixture was purified with chromatography plates using ethyl acetate∶n-hexane (2:4) as the eluent. It is noteworthy that in all of the reactions, the starting materials were not consumed completely, even with an increase in the reaction times to 48 h.

# IR, ^1^H NMR, ^13^C NMR spectrum analyses of products


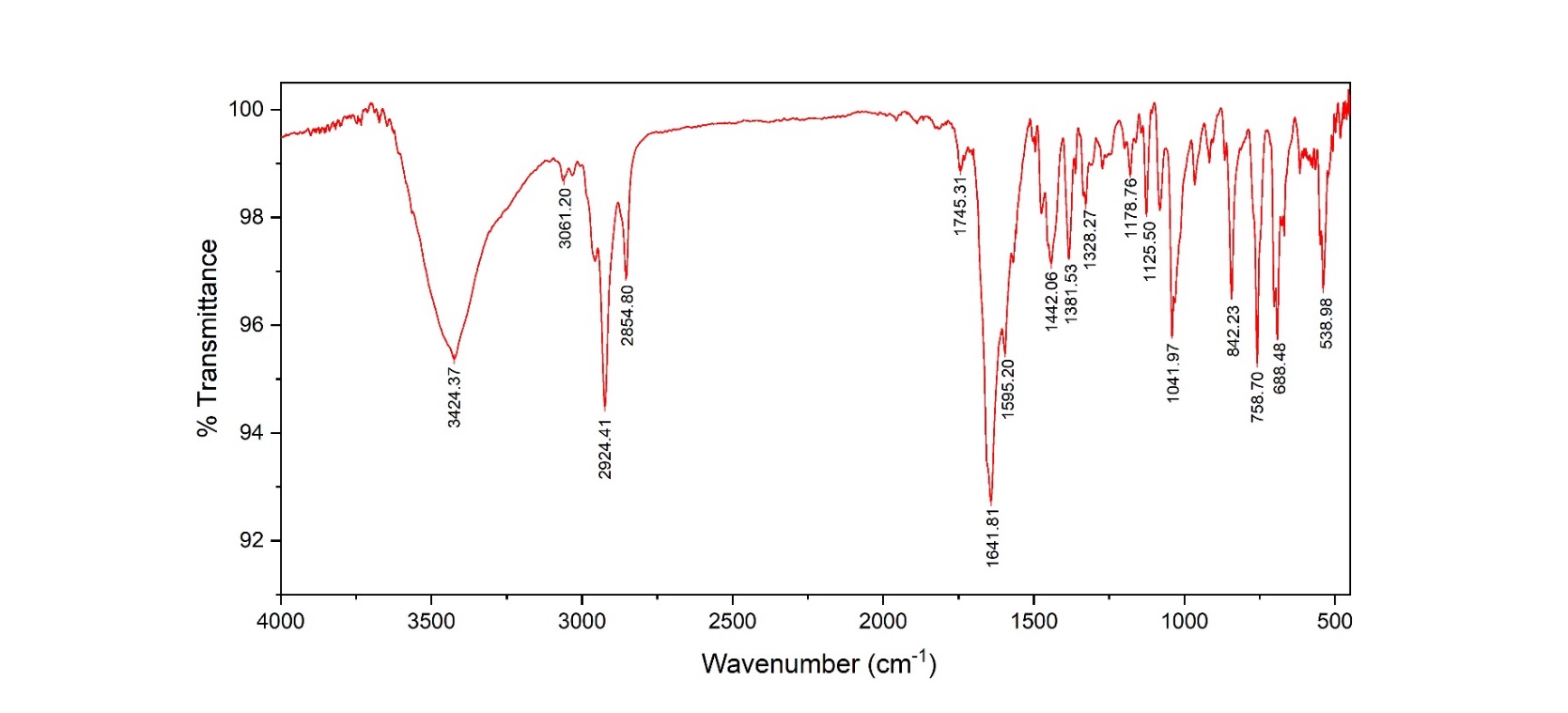


## Figure S1: FT-IR (KBr) spectrum of 7a


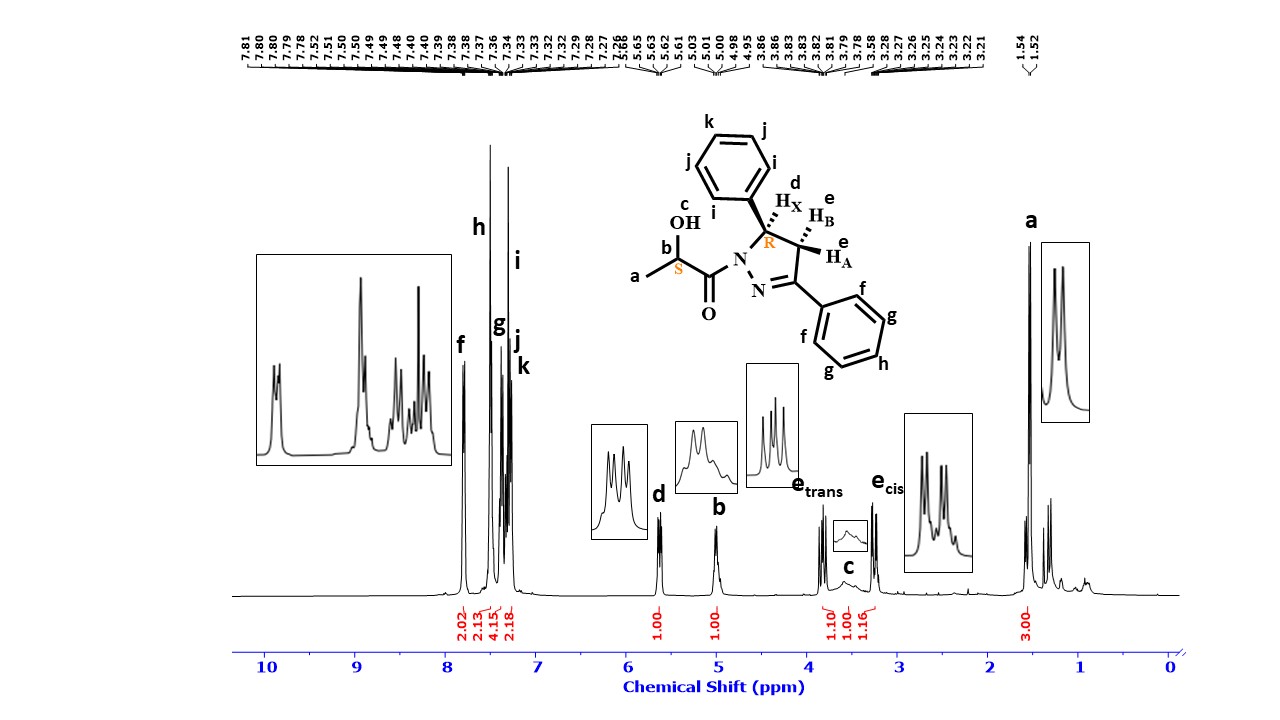


## Figure S2: ^1^H NMR (400 MHz, CDCl_3_) spectrum of 7a


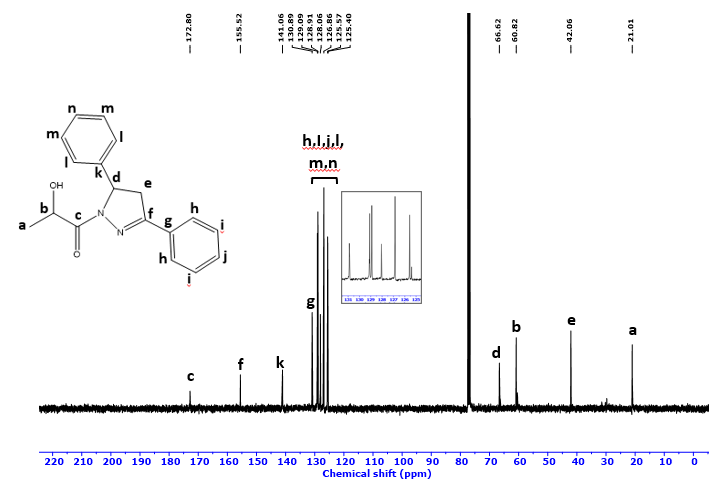


## Figure S3:^13^C NMR (101 MHz, CDCl_3_) spectrum of 7a


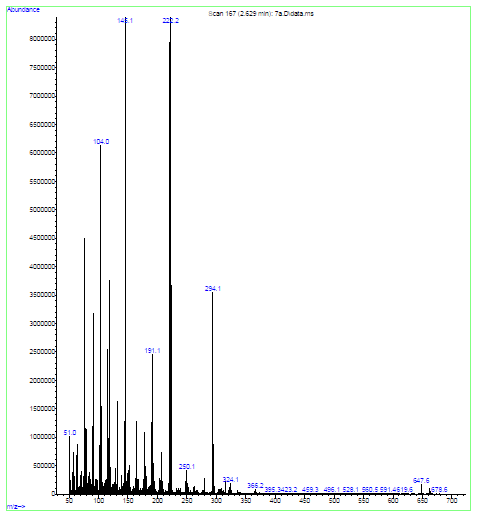


M^+^= 294.1

## Figure S4. Mass spectrum of 7a


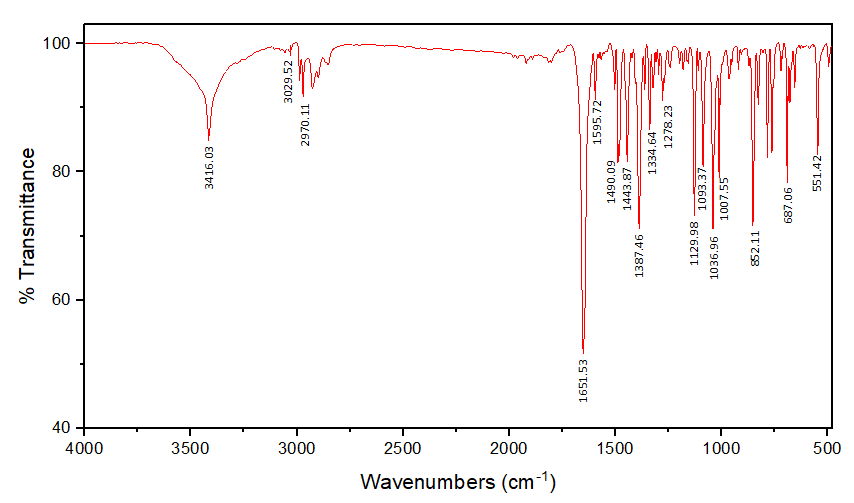


## Figure S5: FT-IR (KBr) spectrum of 7b


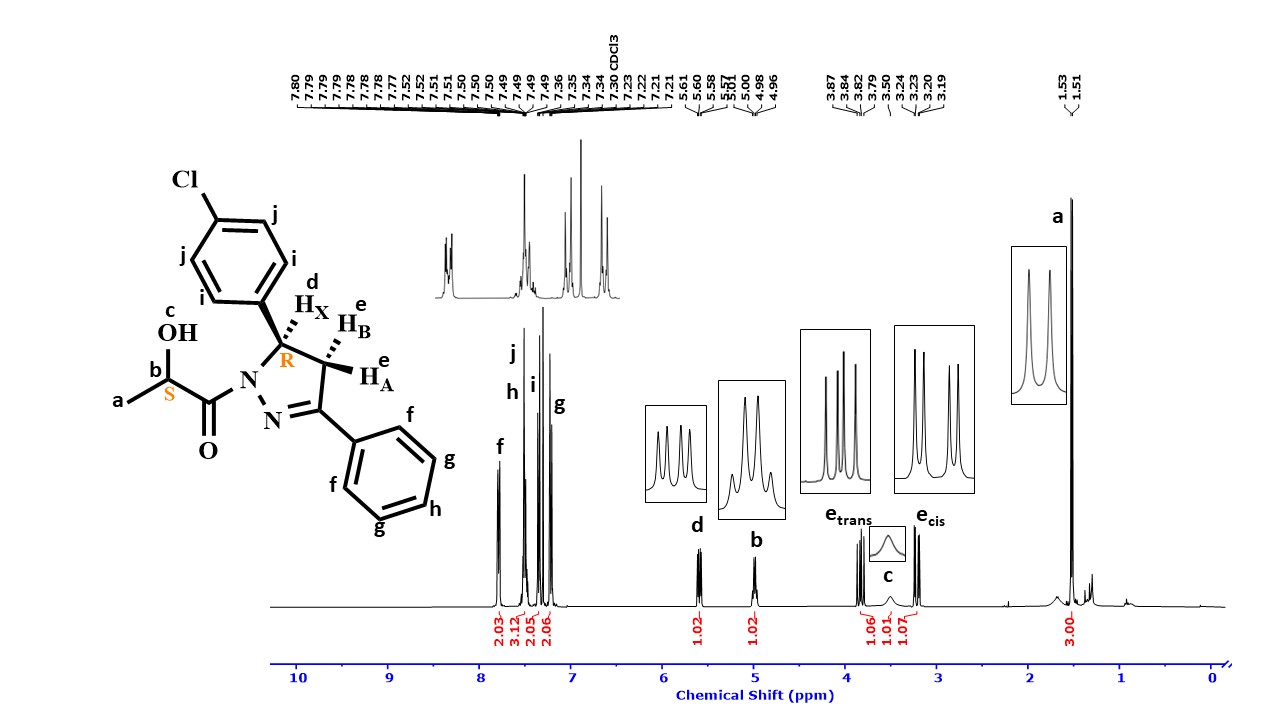


## Figure S6: ^1^H NMR (400 MHz, CDCl_3_) spectrum of 7b


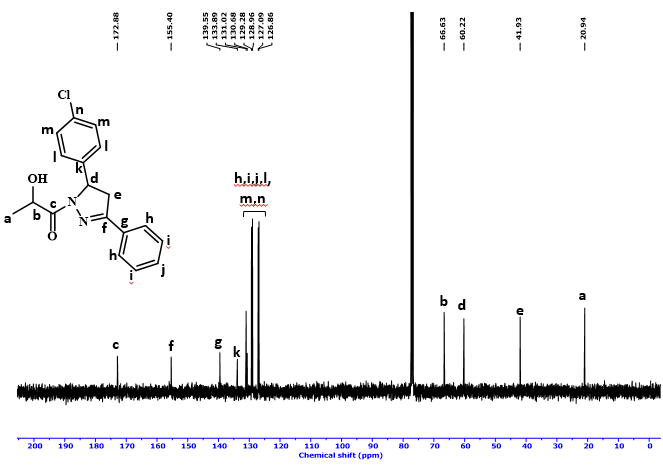


## Figure S7: ^13^C NMR (101 MHz, CDCl_3_) spectrum of 7b


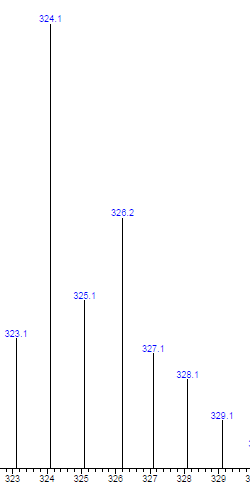

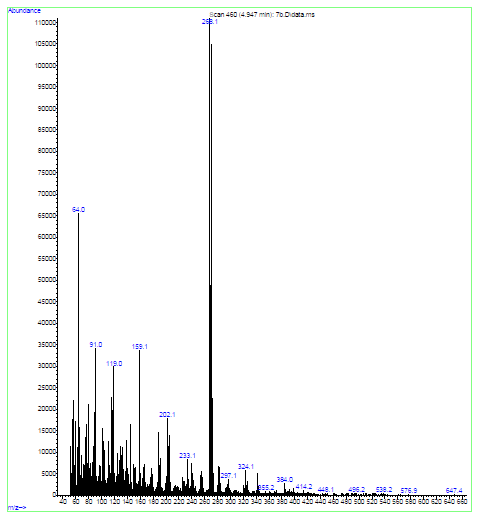


M^+^= 328.1

## Figure S8. Mass spectrum of 7b


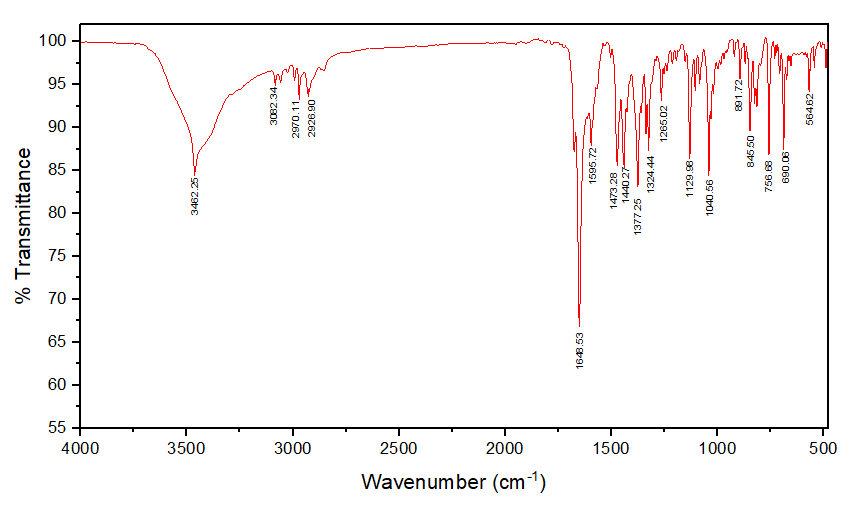


## Figure S9: FT-IR (KBr) spectrum of 7c


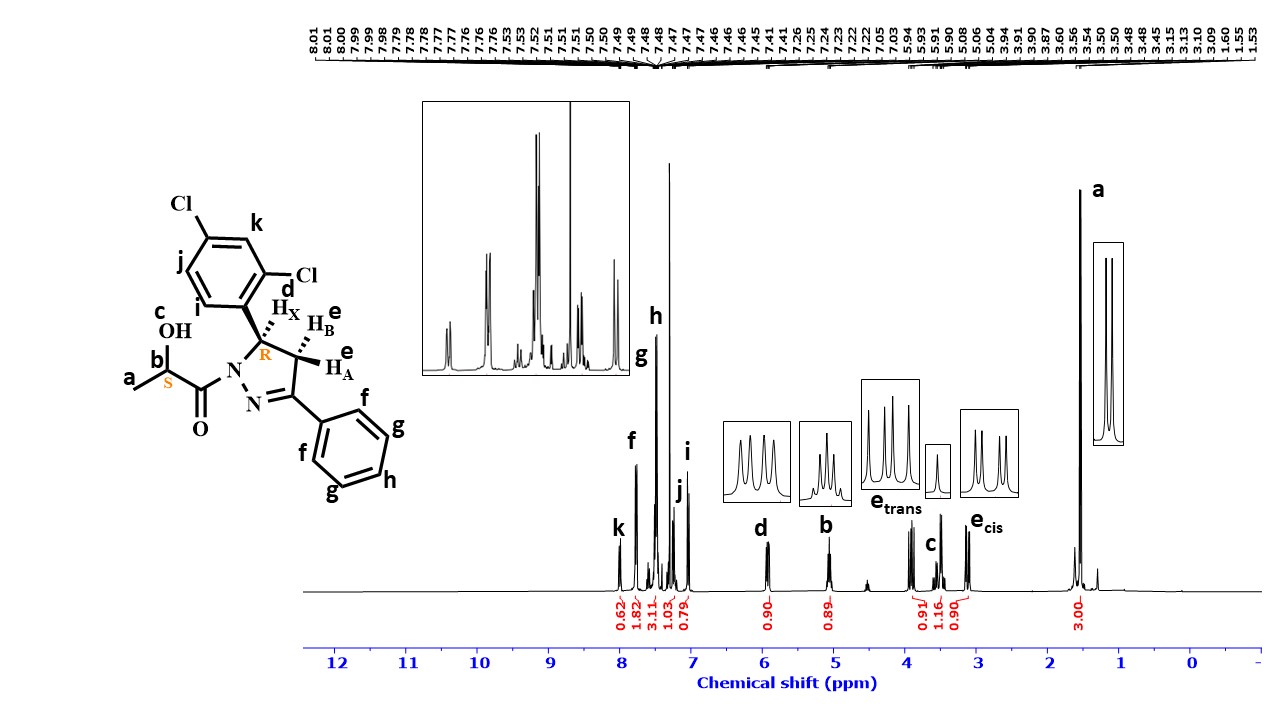


## Figure S10: ^1^H NMR (400 MHz, CDCl_3_) spectrum of 7c


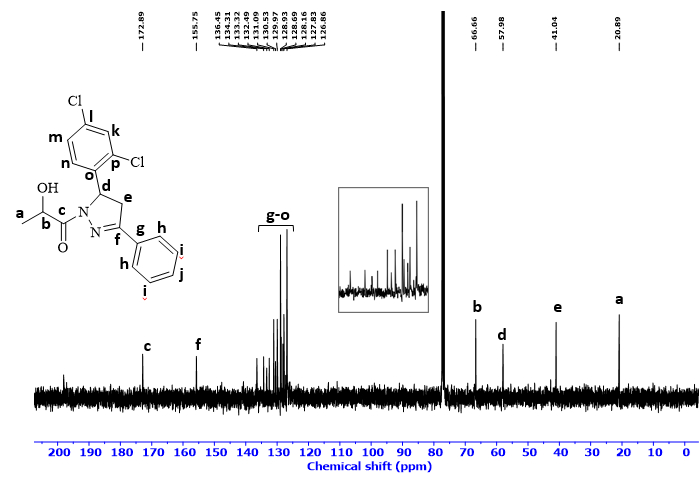


## Figure S11: ^13^C NMR (101 MHz, CDCl_3_) spectrum of 7c


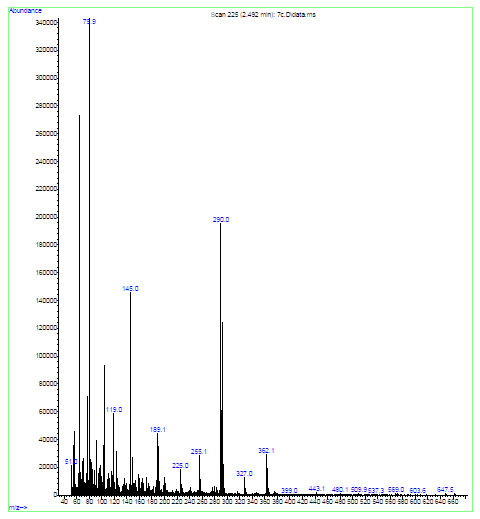


M^+^= 363.1

## Figure S12. Mass spectrum of 7c


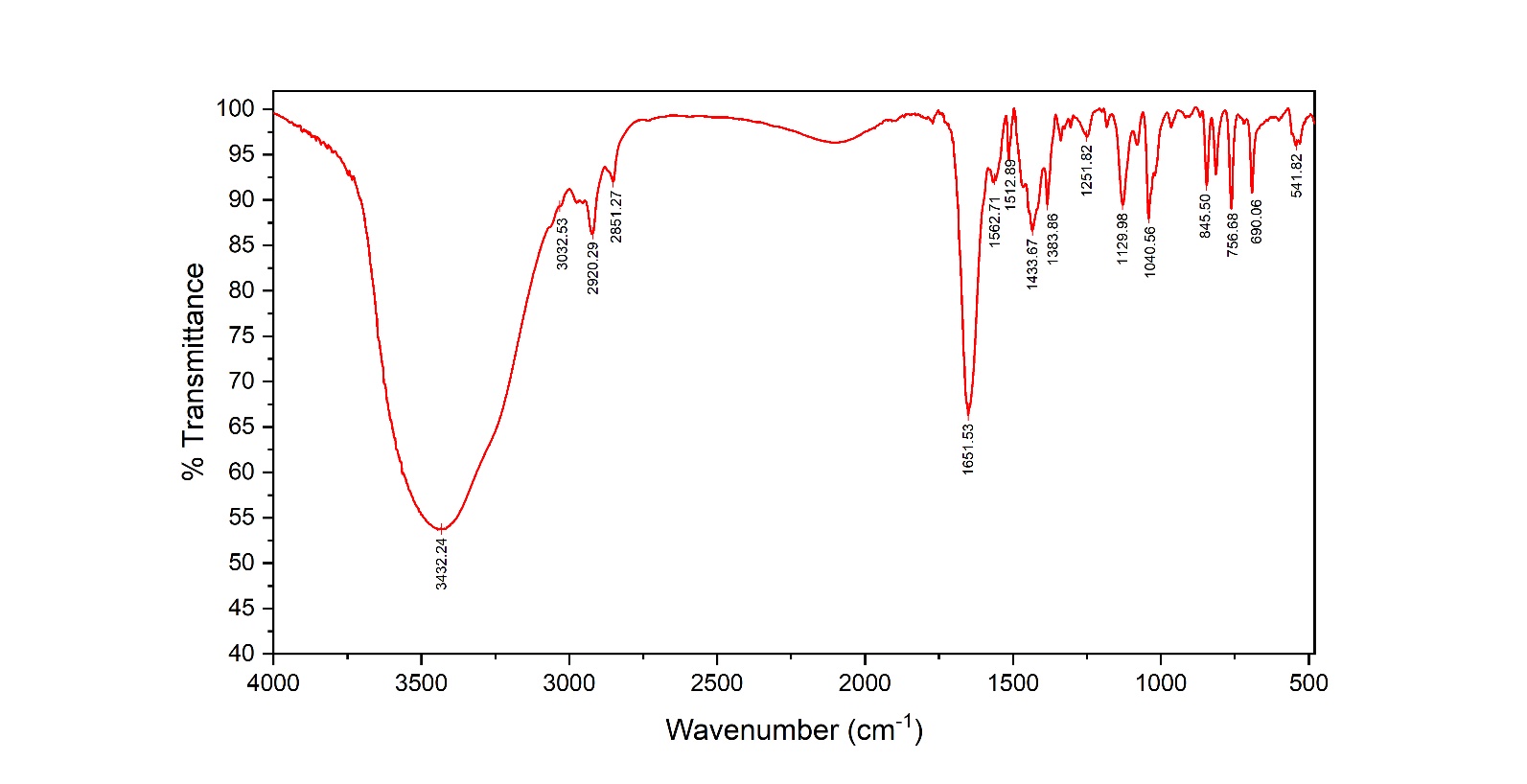


##

## Figure S13: FT-IR (KBr) spectrum of 7d


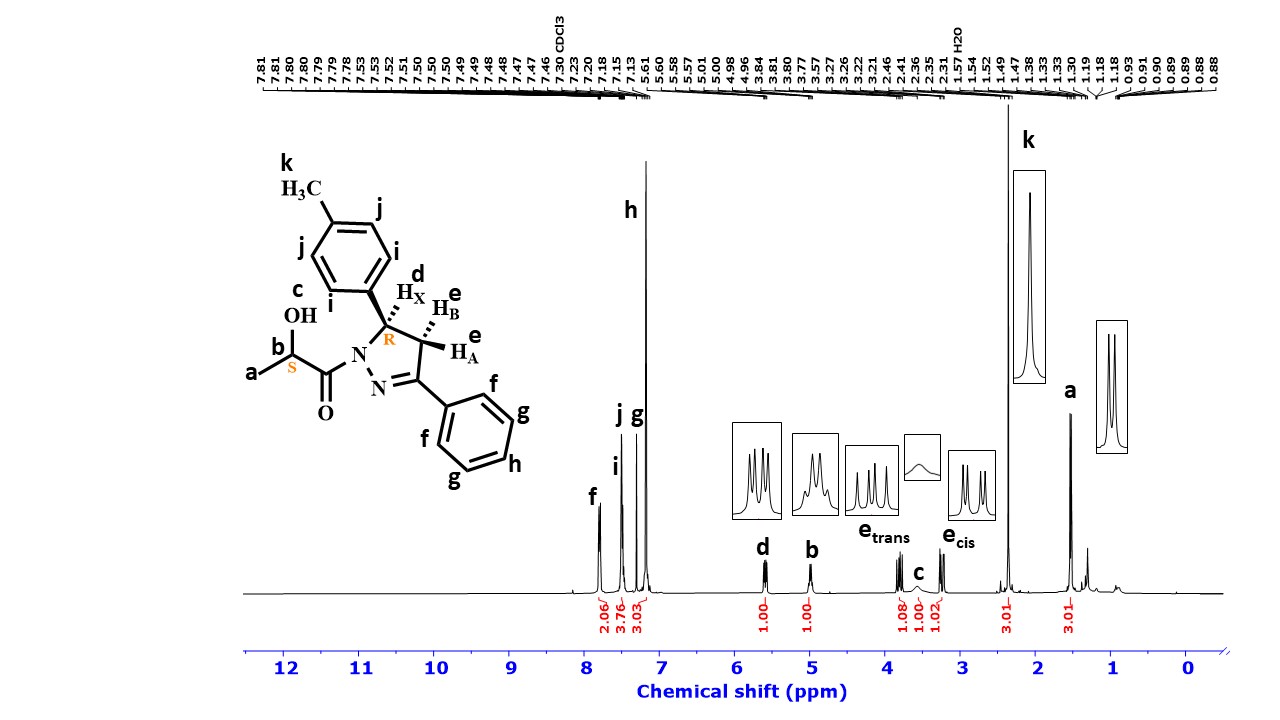


## Figure S14: ^1^H NMR (400 MHz, CDCl_3_) spectrum of 7d


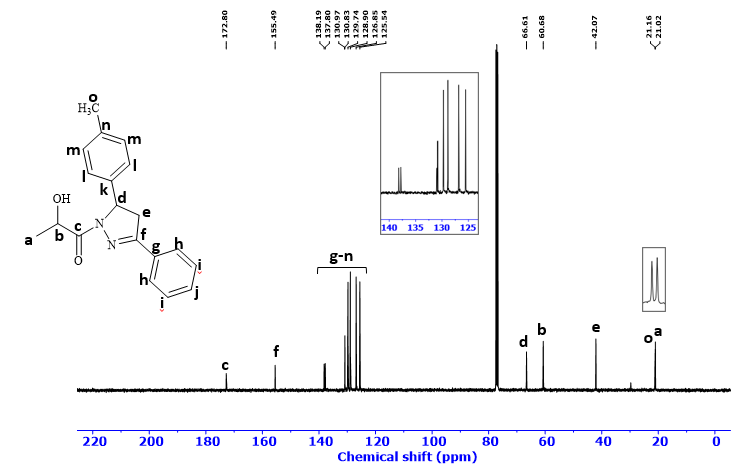


## Figure S15: ^13^C NMR (101 MHz, CDCl_3_) spectrum of 7d


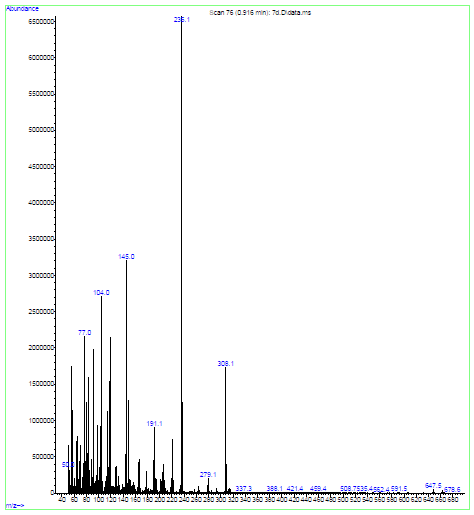


M^+^= 308.1

## Figure S16. Mass spectrum of 7d


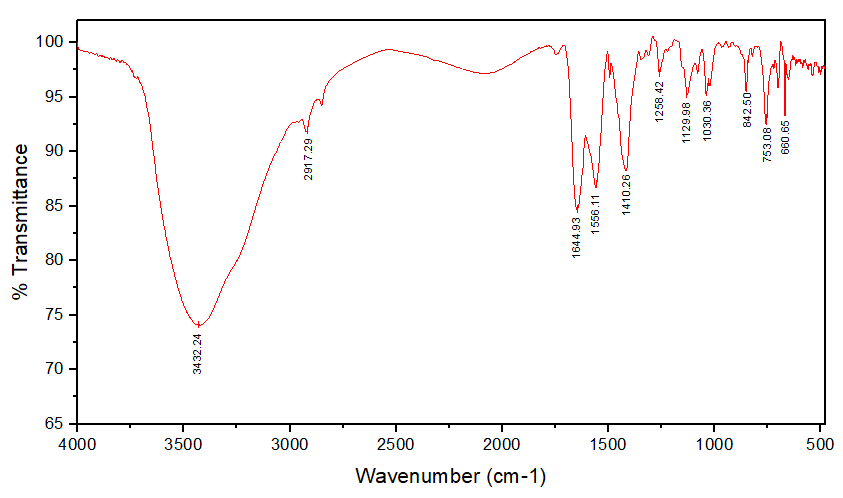


## Figure S17: FT-IR (KBr) spectrum of 7e


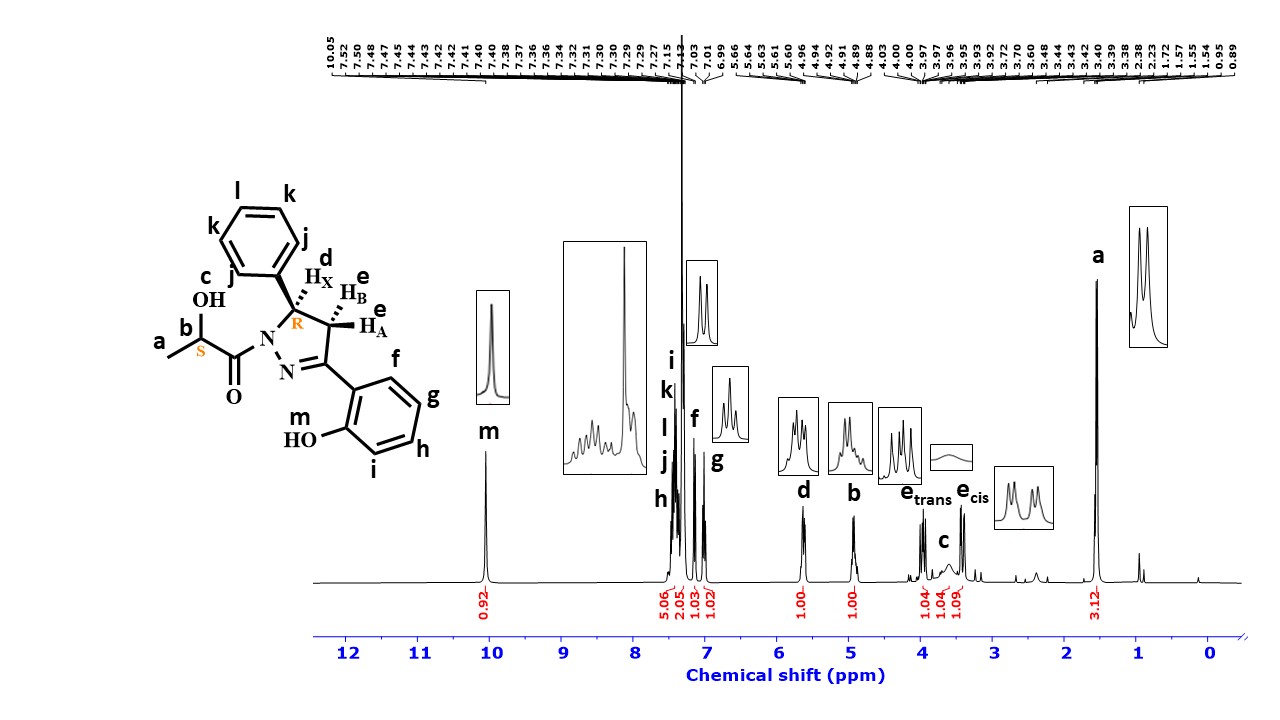


## Figure S18: ^1^H NMR (400 MHz, CDCl_3_) spectrum of 7e


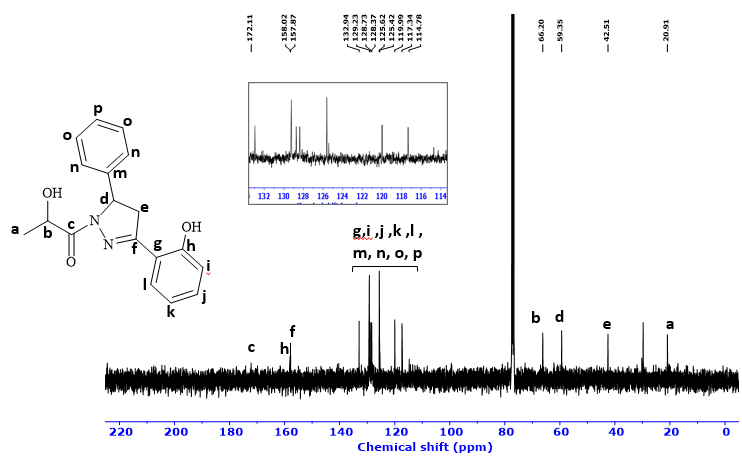


## Figure S19: 13C NMR (CDCl3, 101 MHz) spectrum of 7e


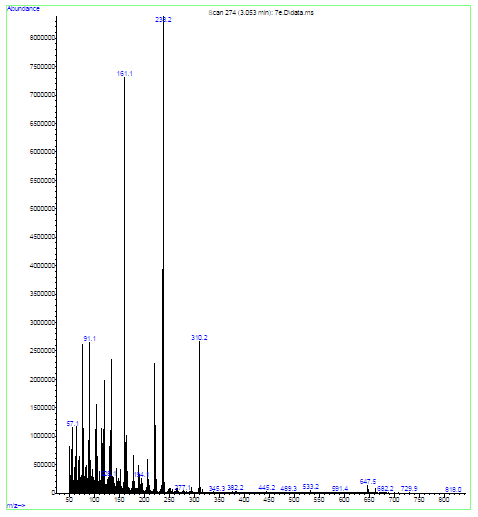


M^+^= 310.2

## Figure S20. Mass spectrum of 7e


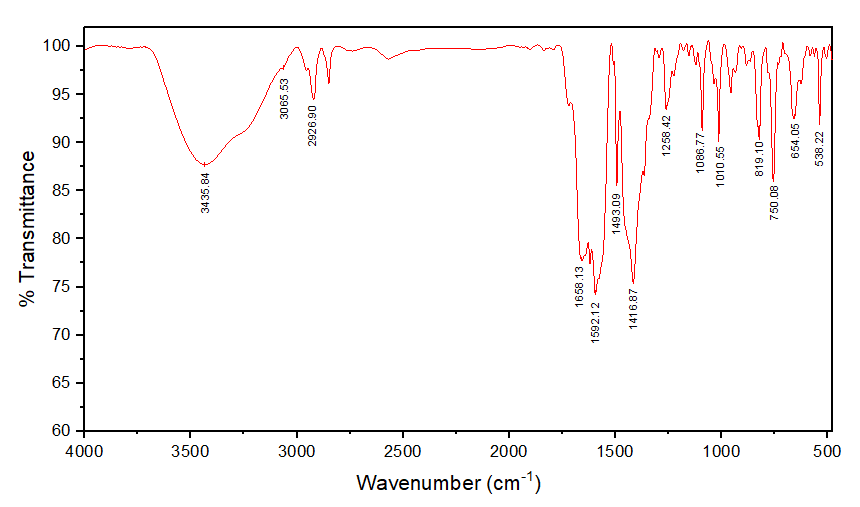


## Figure S21: FT-IR (KBr) spectrum of 7f


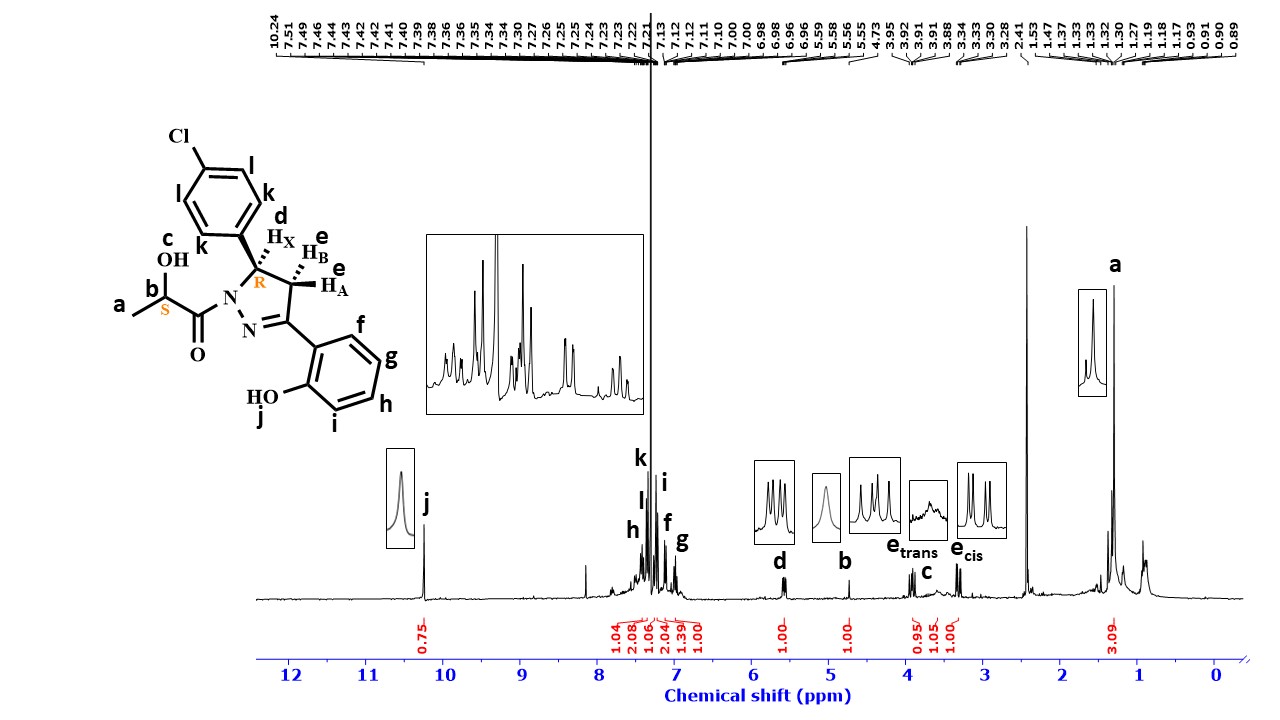


## Figure S22: ^1^H NMR (400 MHz, CDCl3) spectrum of 7f


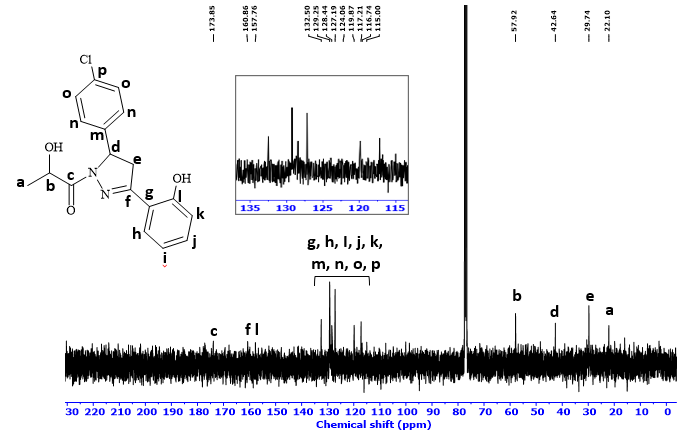


## Figure S23: ^13^C NMR (CDCl_3_, 101 MHz) spectrum of 7f


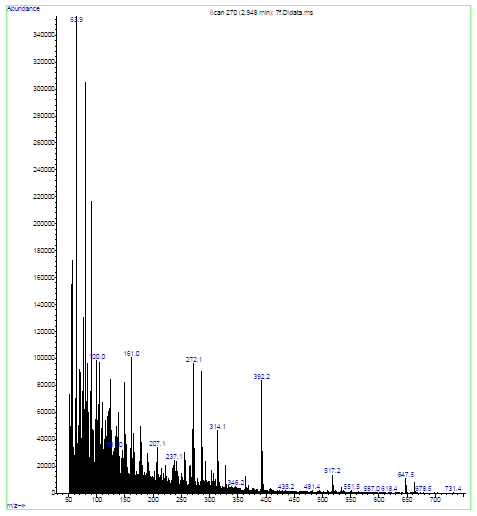


M^+^= 344.1

## Figure S24. Mass spectrum of 7f


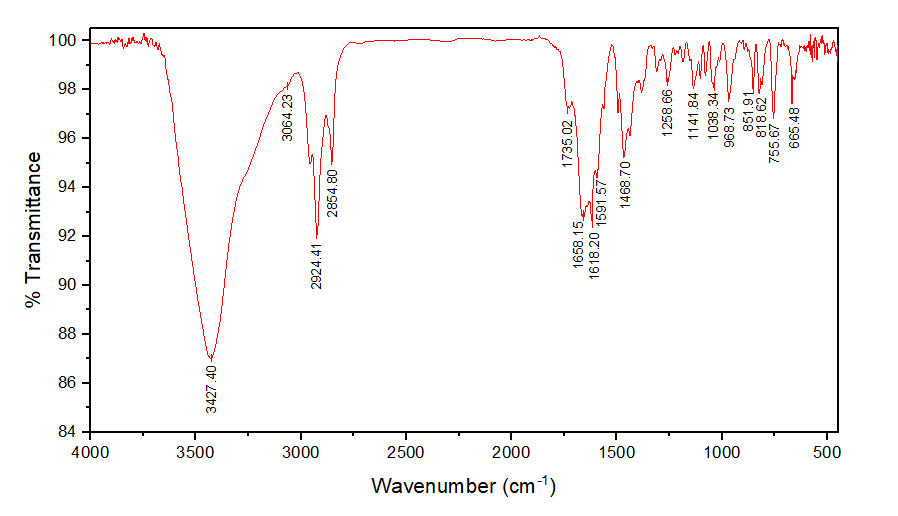


## Figure S25: FT-IR (KBr) spectrum of 7g


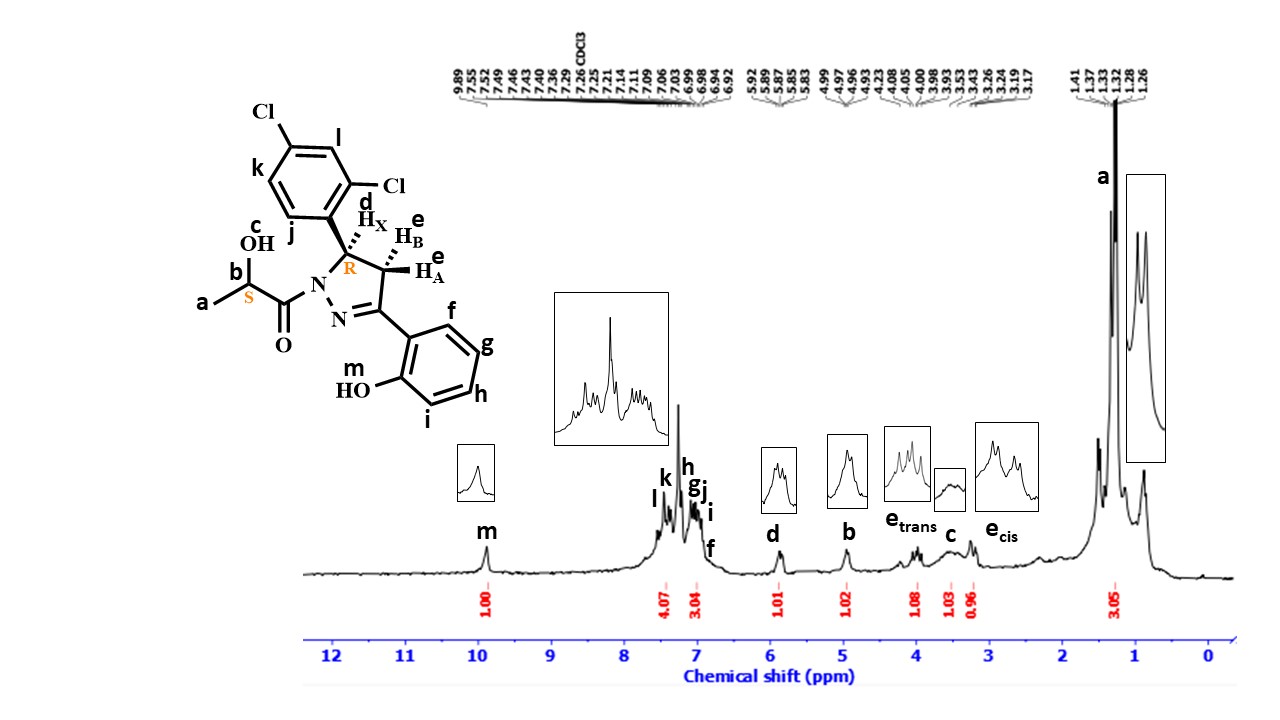


## Figure S26: ^1^H NMR (250 MHz, CDCl_3_) spectrum of 7g


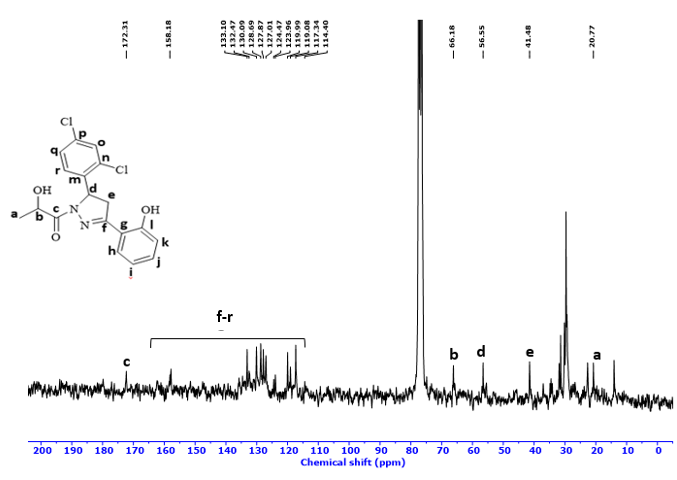


## Figure S27: ^13^C NMR (63 MHz, CDCl_3_) spectrum of 7g


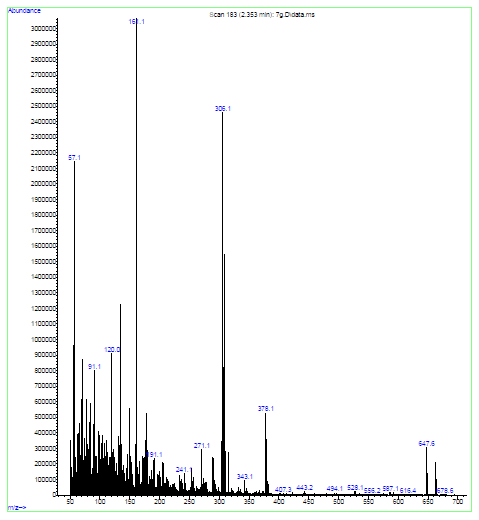


M^+^= 379.1

## Figure S28. Mass spectrum of 7g


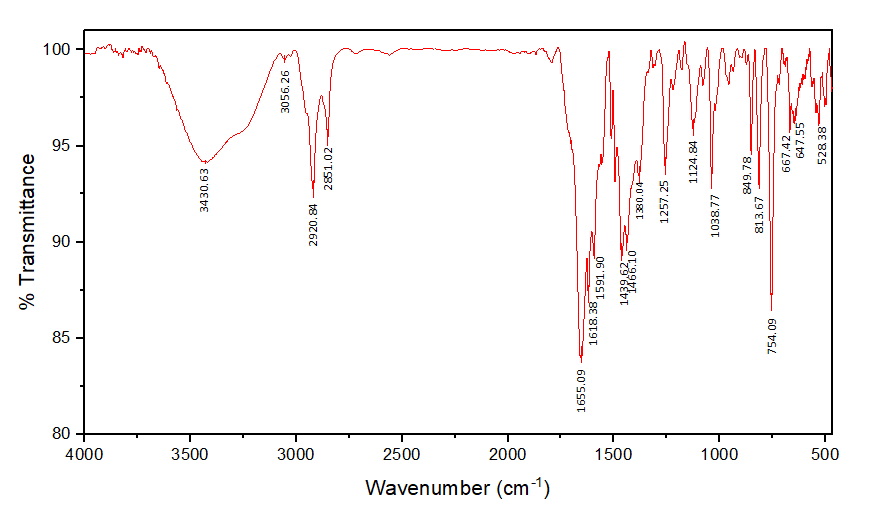


## Figure S29: FT-IR (KBr) spectrum of 7h


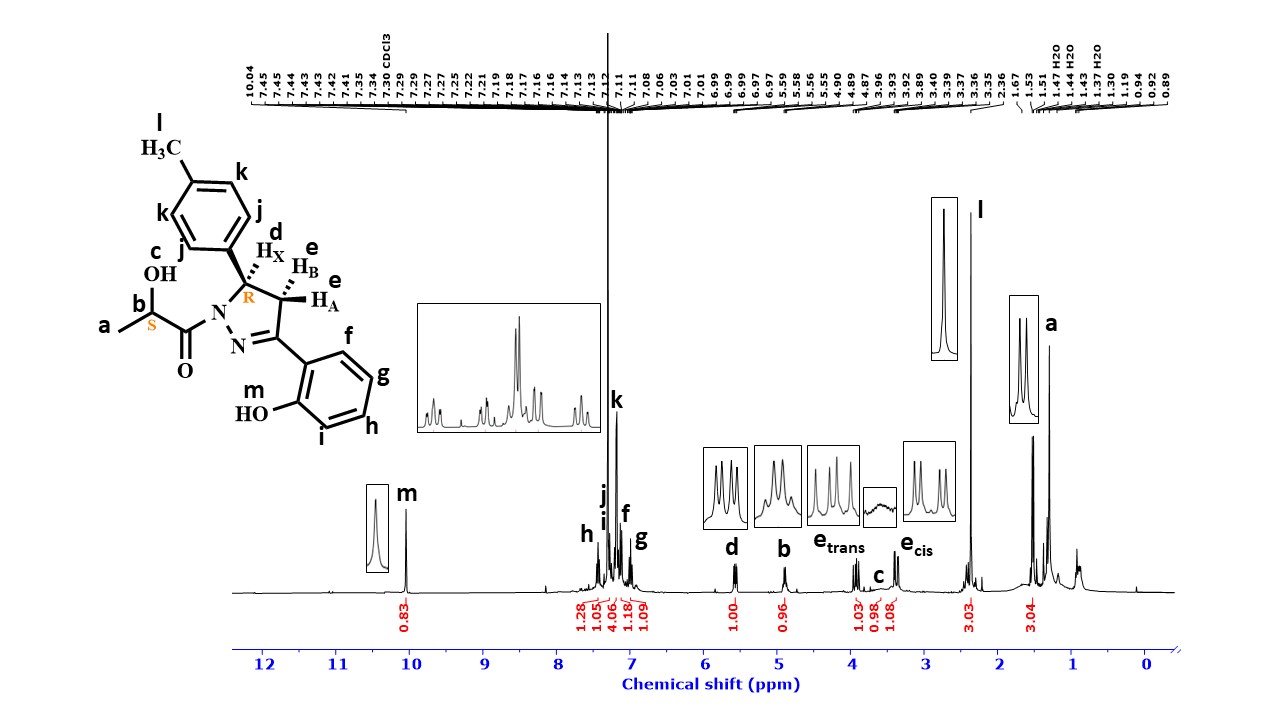


## Figure S30: ^1^H NMR (400 MHz, CDCl_3_) spectrum of 7h


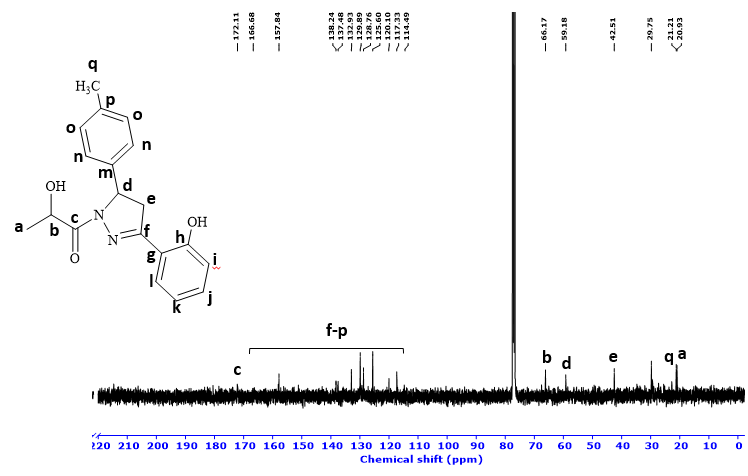


## Figure S31: ^13^C NMR (101 MHz, CDCl_3_) spectrum of 7h


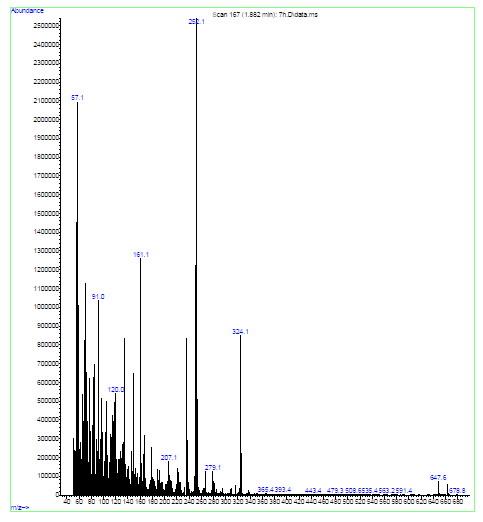


M^+^= 324.1

## Figure S32. Mass spectrum of 7h


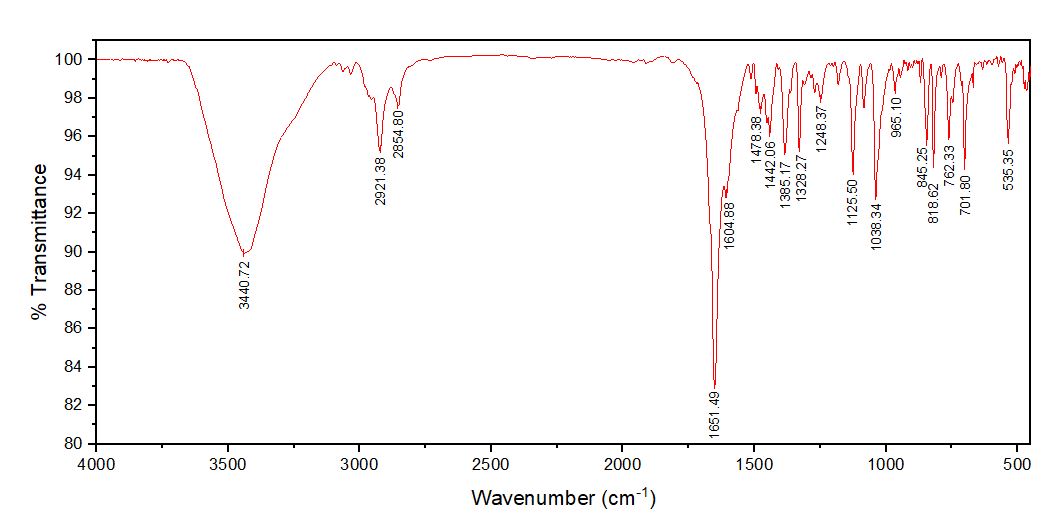


**طیف کربن overnight میخواد**

## Figure S33: FT-IR (KBr) spectrum of 7i


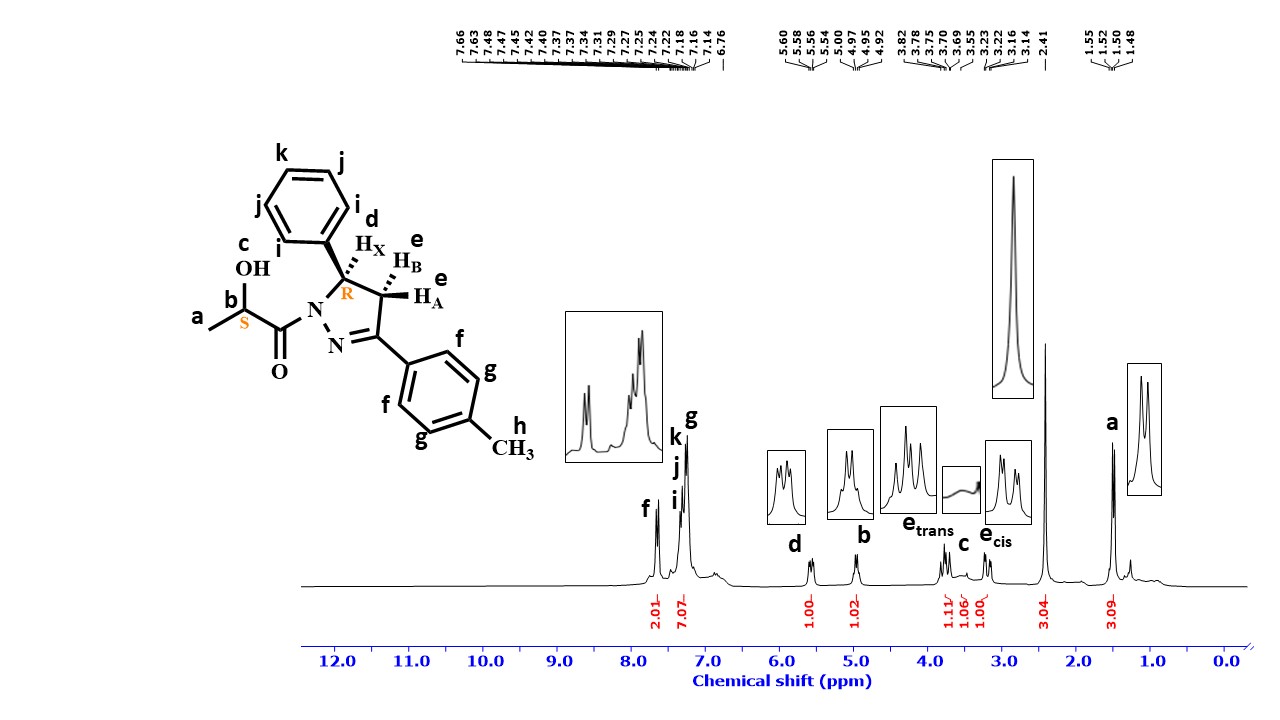


## Figure S34: ^1^H NMR (250 MHz, CDCl_3_) spectrum of 7i


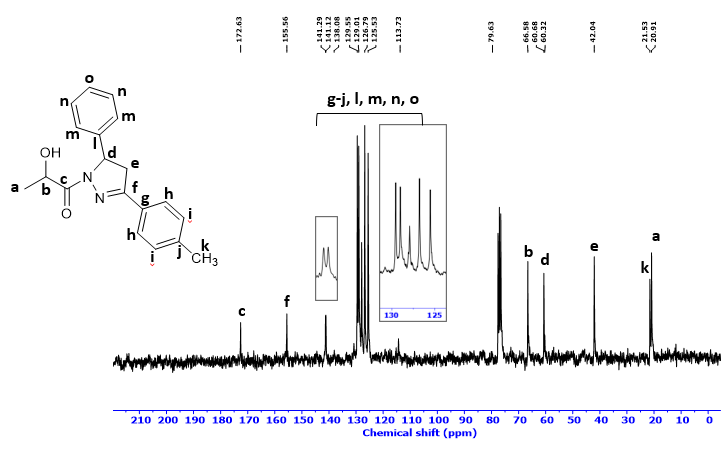


## Figure S35: ^13^C NMR (63 MHz, CDCl_3_) spectrum of 7i


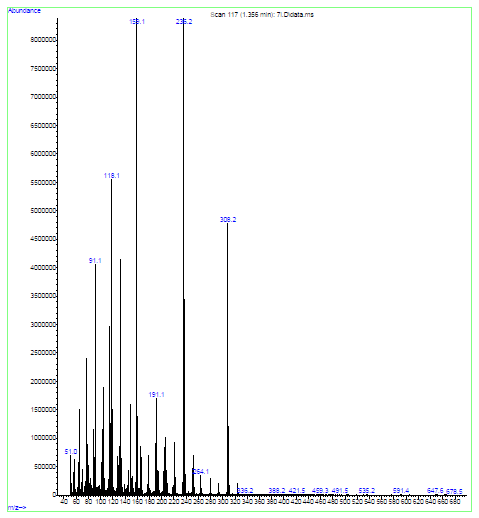


M^+^= 308.1

## Figure S36. Mass spectrum of 7i


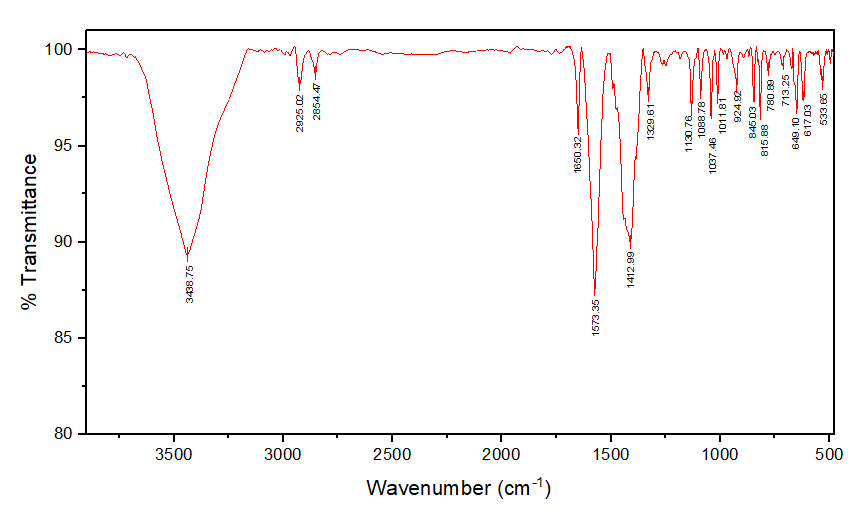


## Figure S37: FT-IR (KBr) spectrum of 7j


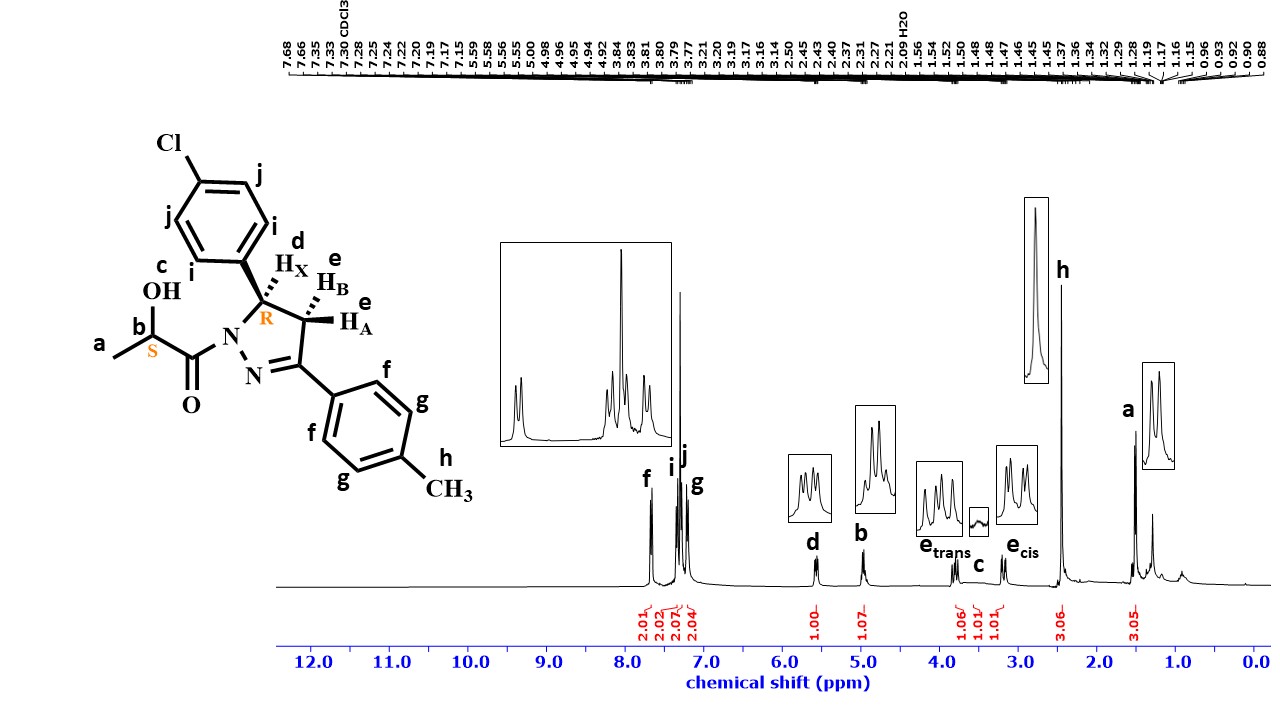


## Figure S38: ^1^H NMR (400 MHz, CDCl_3_) spectrum of 7j


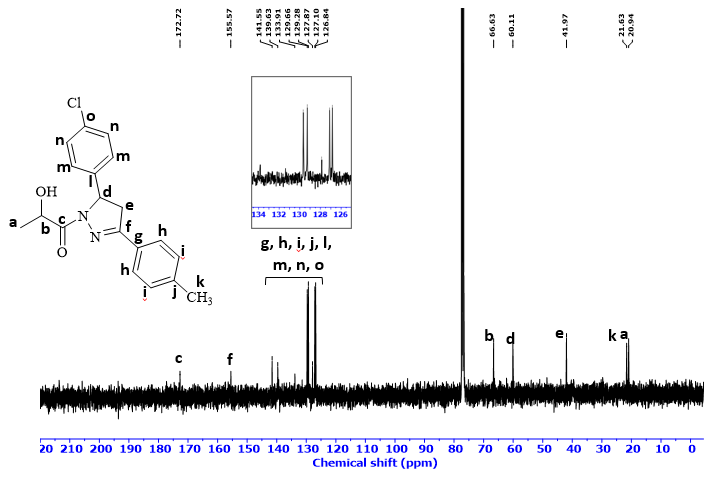


## Figure S39: ^13^C NMR (101 MHz, CDCl_3_) spectrum of 7j


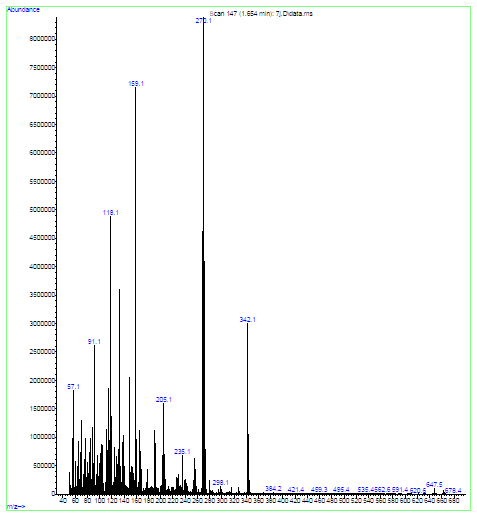


M^+^= 342.1

## Figure S40. Mass spectrum of 7j


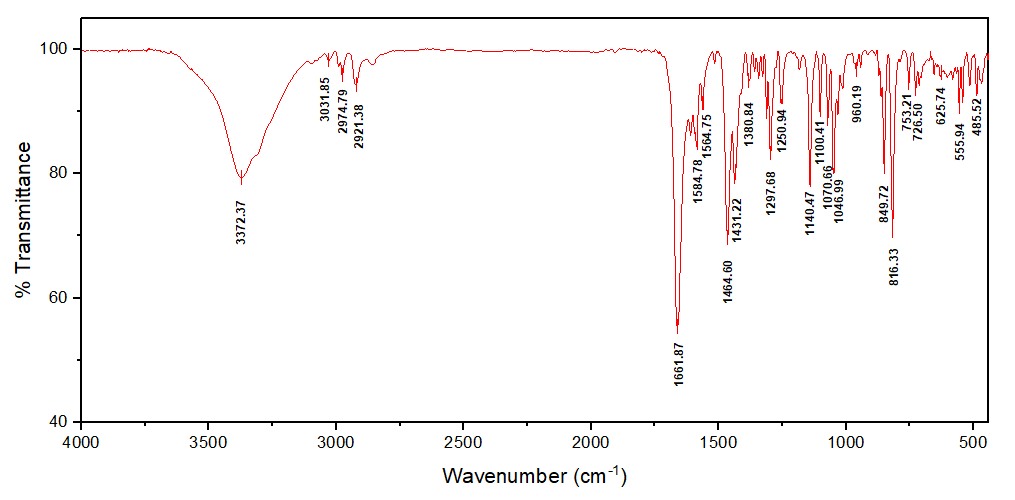


## Figure S41: FT-IR (KBr) spectrum of 7k


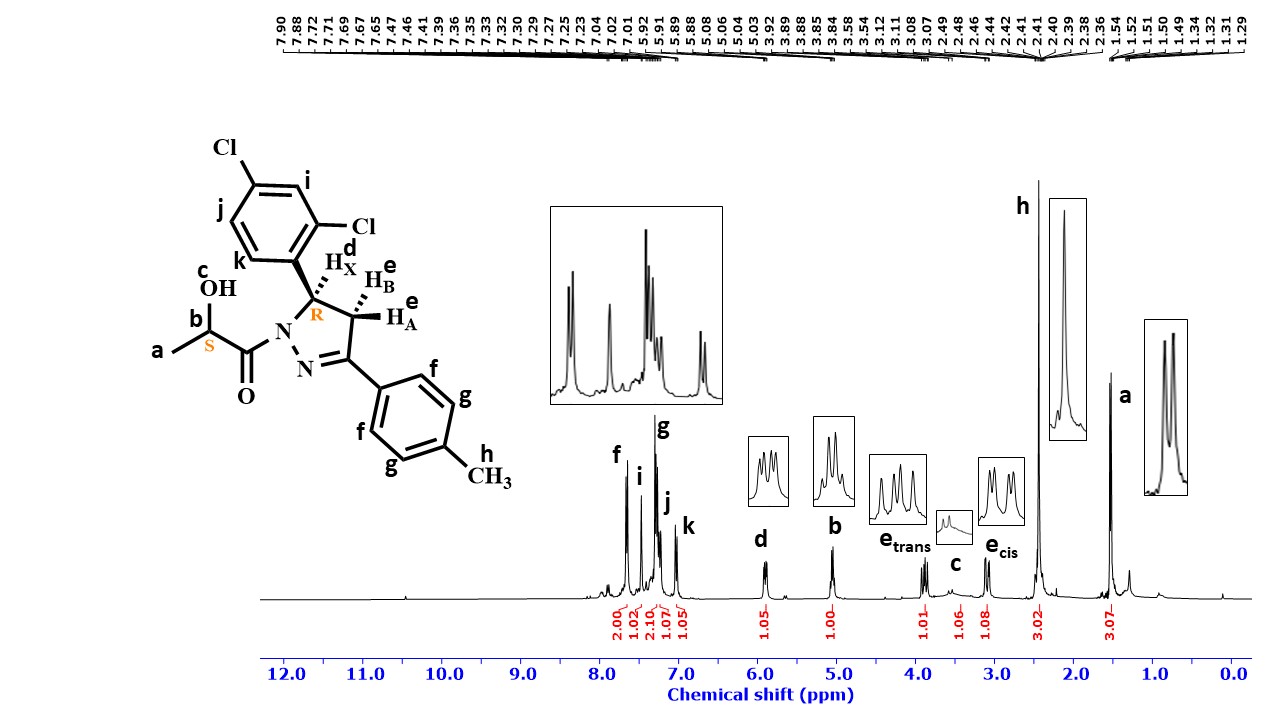


## Figure S42: ^1^H NMR (400 MHz, CDCl_3_) spectrum of 7k


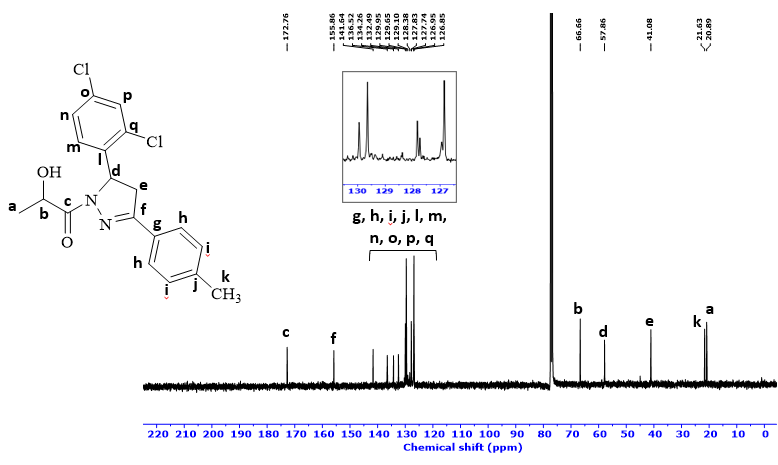


## Figure S43: ^13^C NMR (101 MHz, CDCl_3_) spectrum of 7k


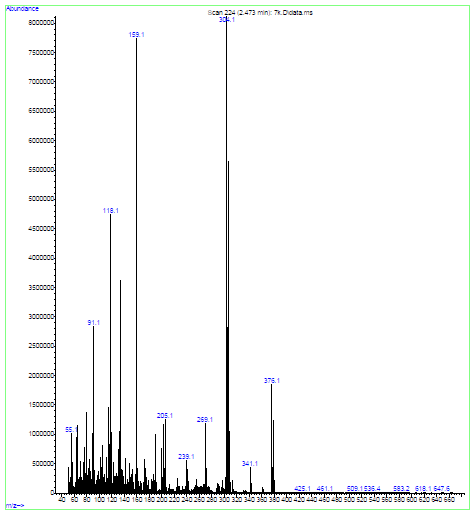


M^+^= 377.1

## Figure S44. Mass spectrum of 7k


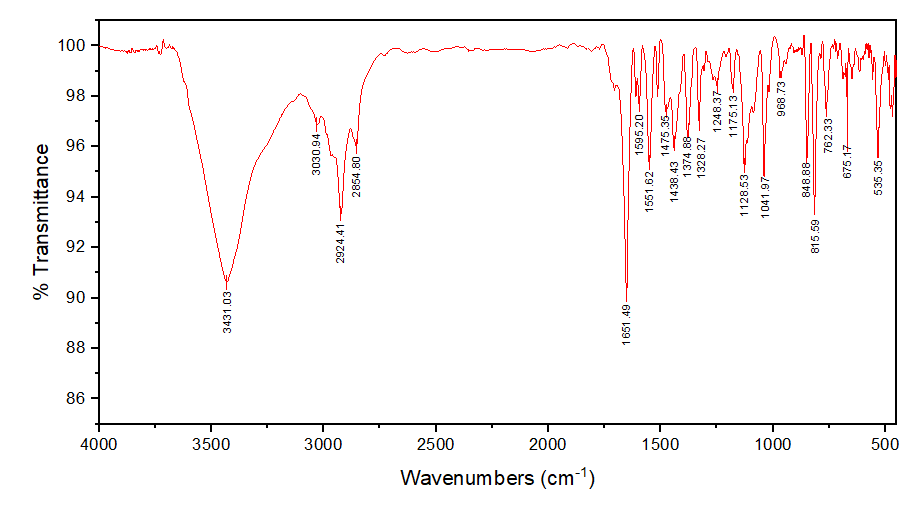


## Figure S45: FT-IR (KBr) spectrum of 7l


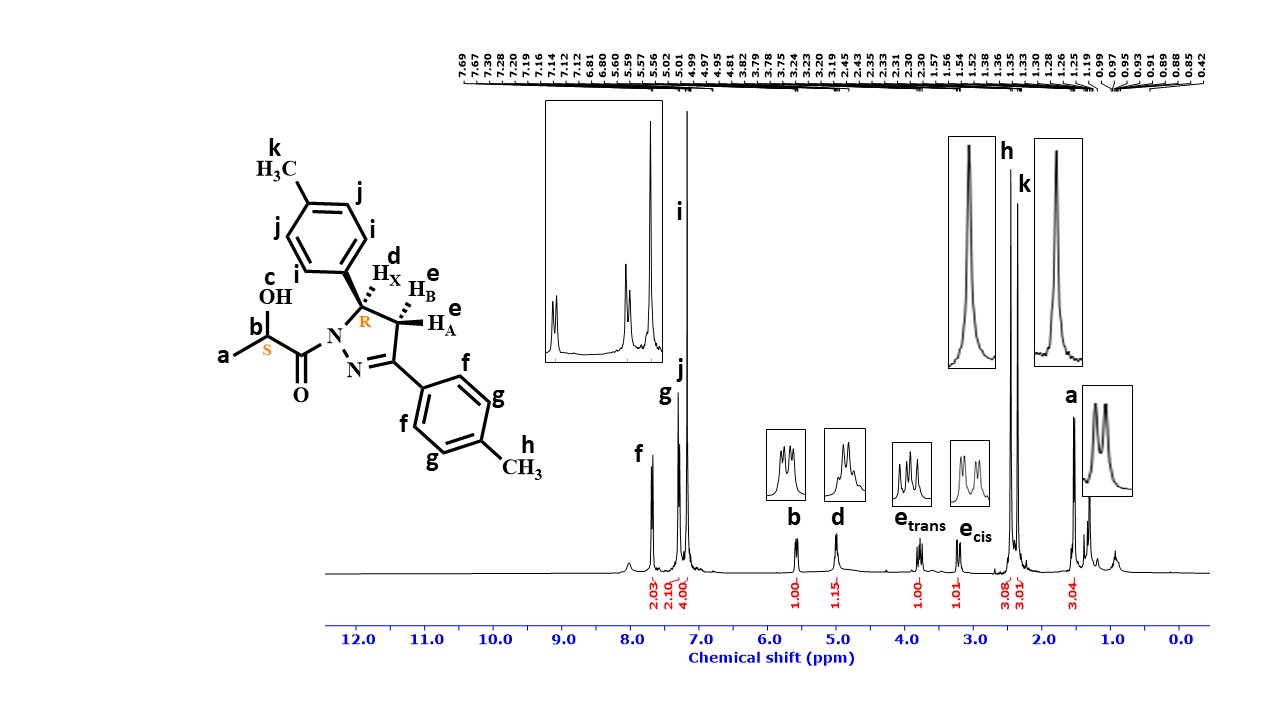


## Figure S46: ^1^H NMR (400 MHz, CDCl_3_) spectrum of 7l


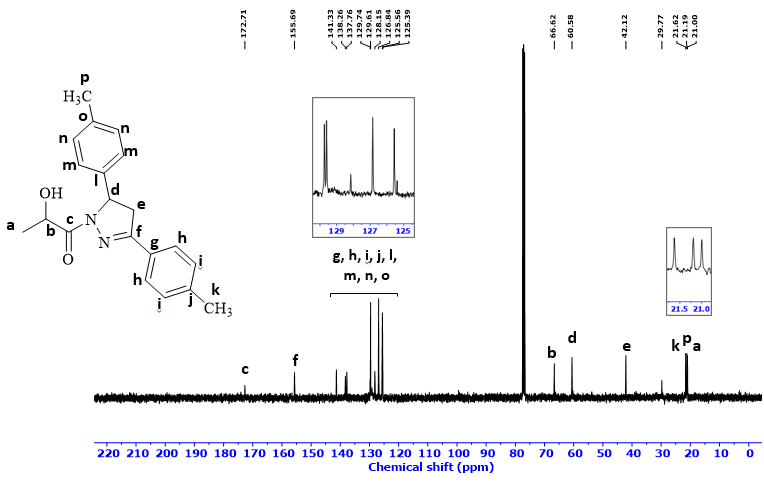


## Figure S47: ^13^C NMR (101 MHz, CDCl_3_) 1spectrum of 7l


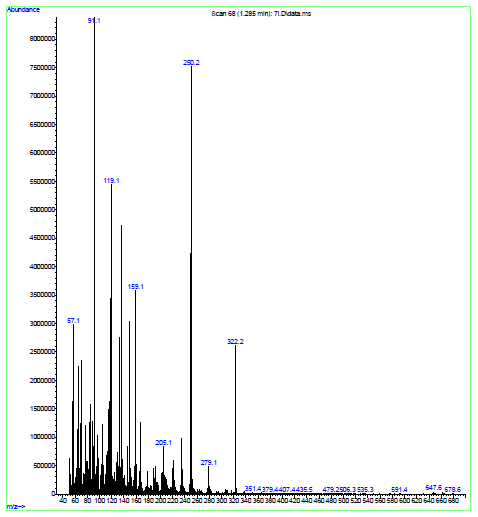


M^+^= 322.2

## Figure S48. Mass spectrum of 7l

# Crystallographic data of compound 7b

X-ray crystallography of lactic-pyrazoline **7b** (CCDC: 2457374) provided clear evidence for the structure of the annulation products.

| 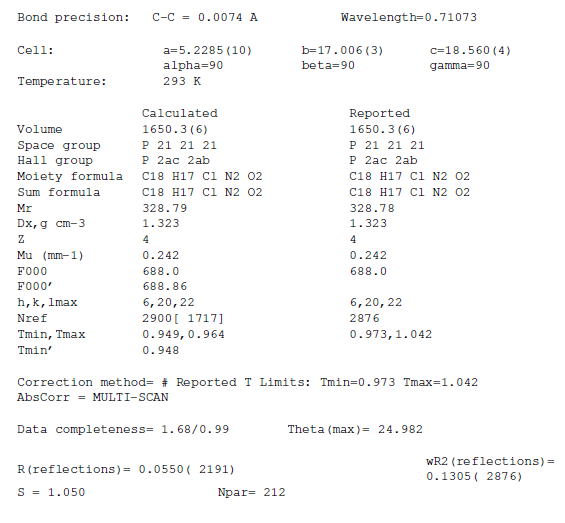 |
| --- |


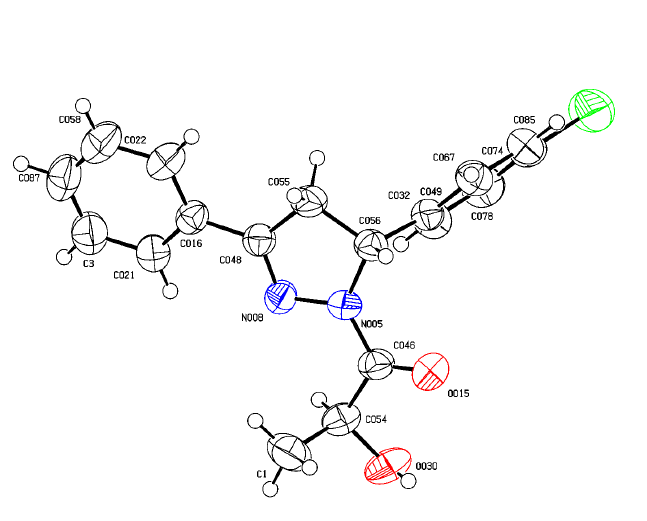


## Figure S49. ORTEP representation of compound **7b**.


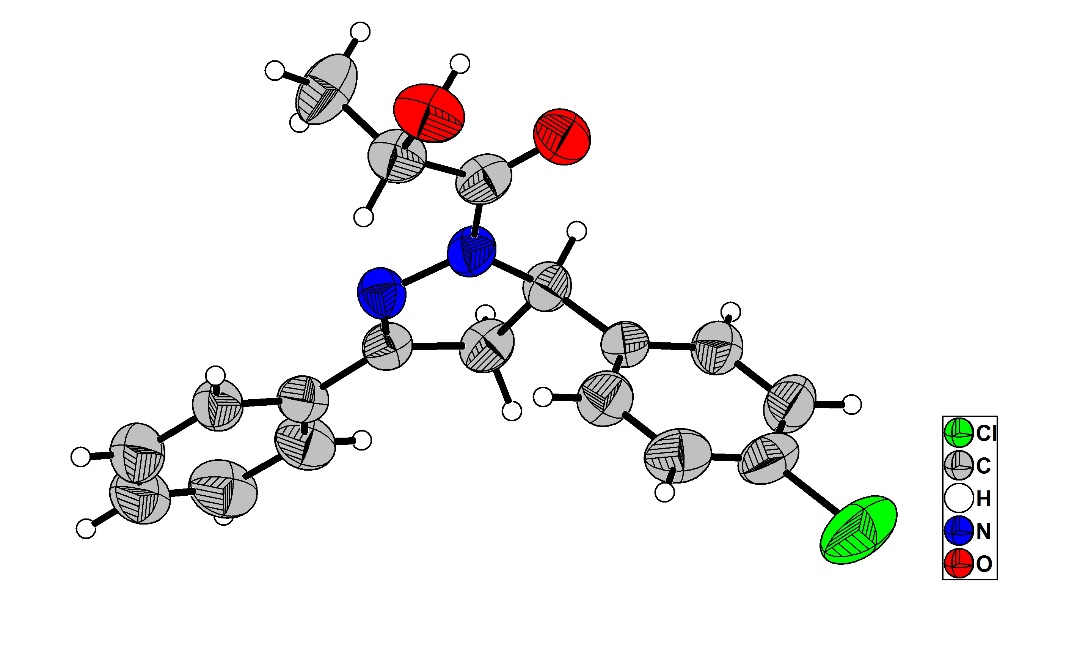


## Figure S50. Crystal structure of compound **7b**

**References**

(1) Noshiranzadeh, N.; Heidari, A.; Haghi, F.; Bikas, R.; Lis, T. Chiral lactic hydrazone derivatives as potential bioactive antibacterial agents: Synthesis, spectroscopic, structural and molecular docking studies. *Journal of Molecular Structure* **2017**, *1128*, 391-399.

(2) Suwito, H.; Jumina; Mustofa; Pudjiastuti, P.; Fanani, M. Z.; Kimata-Ariga, Y.; Katahira, R.; Kawakami, T.; Fujiwara, T.; Hase, T. Design and synthesis of chalcone derivatives as inhibitors of the ferredoxin—Ferredoxin-NADP+ reductase interaction of plasmodium falciparum: Pursuing new antimalarial agents. *Molecules* **2014**, *19* (12), 21473-21488.

(3) Akhtar, M. J.; Khan, A. A.; Ali, Z.; Dewangan, R. P.; Rafi, M.; Hassan, M. Q.; Akhtar, M. S.; Siddiqui, A. A.; Partap, S.; Pasha, S. Synthesis of stable benzimidazole derivatives bearing pyrazole as anticancer and EGFR receptor inhibitors. *Bioorganic Chemistry* **2018**, *78*, 158-169.
